# Genomic variations associated with attenuation in *Mycobacterium avium* subsp. *paratuberculosis* vaccine strains

**DOI:** 10.1186/1471-2180-13-11

**Published:** 2013-01-22

**Authors:** Tim J Bull, Alex Schock, J Michael Sharp, Mandisa Greene, Iain J McKendrick, Jill Sales, Richard Linedale, Karen Stevenson

**Affiliations:** 1 St. George’s University of London Medical School, SW17 0RE, London, UK; 2 AHVLA Lasswade, Bush Loan, EH26 0PZ, Penicuik, Midlothian, UK; 3 Moredun Research Institute, Bush Loan, EH26 0PZ, Penicuik, Midlothian, UK; 4 Biomathematics & Statistics Scotland, James Clerk Maxwell Building, The King’s Buildings, Mayfield Road, EH9 3JZ, Edinburgh, UK; 5 Present address: Charter Veterinary Surgeons, 51 Congleton Road, ST8 6EF, Stoke On Trent, UK

**Keywords:** *Mycobacterium avium* subspecies *paratuberculosis*, Vaccine, Comparative genomics, Variable genomic island, Attenuation, Microarray

## Abstract

**Background:**

*Mycobacterium avium* subspecies *paratuberculosis* (MAP) whole cell vaccines have been widely used tools in the control of Johne’s disease in animals despite being unable to provide complete protection. Current vaccine strains derive from stocks created many decades ago; however their genotypes, underlying mechanisms and relative degree of their attenuation are largely unknown.

**Results:**

Using mouse virulence studies we confirm that MAP vaccine strains 316 F, II and 2e have diverse but clearly attenuated survival and persistence characteristics compared with wild type strains. Using a pan genomic microarray we characterise the genomic variations in a panel of vaccine strains sourced from stocks spanning over 40 years of maintenance. We describe multiple genomic variations specific for individual vaccine stocks in both deletion (26–32 Kbp) and tandem duplicated (11–40 Kbp) large variable genomic islands and insertion sequence copy numbers. We show individual differences suitable for diagnostic differentiation between vaccine and wild type genotypes and provide evidence for functionality of some of the deleted MAP-specific genes and their possible relation to attenuation.

**Conclusions:**

This study shows how culture environments have influenced MAP genome diversity resulting in large tandem genomic duplications, deletions and transposable element activity. In combination with classical selective systematic subculture this has led to fixation of specific MAP genomic alterations in some vaccine strain lineages which link the resulting attenuated phenotypes with deficiencies in high reactive oxygen species handling.

## Background

*Mycobacterium avium* subspecies *paratuberculosis* (MAP) is a proven enteric pathogen with a wide host range that includes many domestic and wild animals [1]. It is the causal agent of Johne’s disease (JD) in animals which is particularly common in countries with significant dairy industries leading to considerable economic losses [2]. MAP can infect, disseminate and persist in humans and has been suggested as a contributory factor in the development of Crohn’s disease [3].

MAP vaccines are a major tool used in the control of JD in animals and can be highly profitable [4]. They have advantages over herd management [5] and culling strategies [6] in being more cost efficient, easier to implement on a wide scale and less reliant on diagnostic testing. It is clear however, that although able to prevent a majority of animals from reaching onset of clinical disease, their current formulations provide incomplete protection against infection and shedding [7-9], thus failing to eradicate the organism [10]. Most current whole cell vaccine preparations rely on subcultures of classic strains that were generated over 70 years ago [11] and some evidence suggests that, for killed preparations at least, more recently acquired local virulent strain types may be more effective [12]. Previous experience with BCG has shown that frequent *in vitro* passage of strains in different laboratories led to significant alterations in genomic profiles and diversities in attenuation and immunogenicity [13]. It is of importance therefore to derive accurate definitions of MAP vaccine genotypes to better standardize vaccine manufacture and understand the critical mechanisms determining vaccine attenuations and protective efficacies.

The distribution and worldwide use of MAP vaccines has continued since live ‘attenuated’ strains were selected in France (1924) and the UK (1940) using a method of sequential passage similar to that applied for the generation of BCG [14]. The degree and mechanism underlying their attenuation however is uncertain as virulence studies were not performed in any detail. Concerns in the 1980’s regarding the use of live vaccine strains because of low shelf life and spread to the environment promoted the use of killed vaccine formulations. These were based on various combinations of three MAP strains comprising strain 2e from the UK, strain II from Canada and 316 F. A combination of these were used as live preparations for Norwegian goats in the 1960’s and 1970’s [15] and as a killed preparation delivered in mineral oil to Icelandic sheep in the 1950’s [16]. Live vaccine formulations of 316 F alone were used in the 1960’s and 70’s in the UK [17] and Cyprus [18], 1980’s in Hungary [19], 1990’s in Germany [20] and Spain [21] and up until 2002 in New Zealand [22]. Killed preparations of 316 F alone have been used extensively worldwide [23] and are still available for commercial use. These strains, due to the difficulty in retaining mycobacteria in frozen seed stocks, have been maintained through regular subculture on a variety of laboratory in-house media. It is unsurprising therefore, that some reports suggest strain adaptation to growth in specialized media with loss of Mycobactin J dependence [24] and genome diversity [25] has occurred amongst some lineages.

In this work we demonstrate attenuation and differential virulence of vaccine strains 2e, II and 316 F in a mouse model and use a full MAP genome microarray, supported by PCR and sequencing to investigate the genomic shifts of vaccine strains from a variety of lineages, including one recently resuscitated 316 F strain, originally lyophilised in 1966. We describe large genomic regions with deletions and tandem duplications uniquely associated with each vaccine clade, demonstrate the functionality of some of these deleted genes and hypothesise as to their role in virulence attenuation.

## Results

### Comparative Genomic Hybridisation of vaccine strains

**Figure 1:**
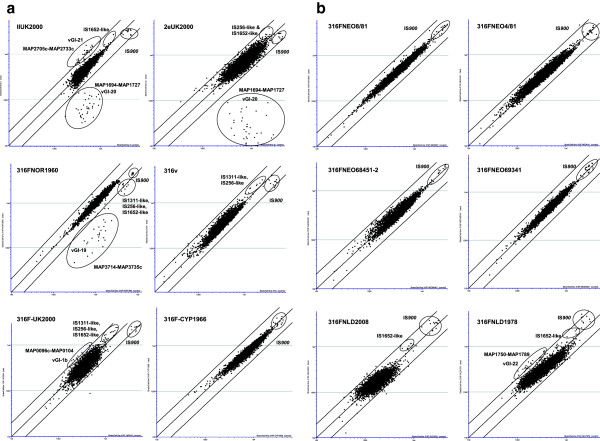
Microarray scatterplots comparing genomes of test MAP vaccine strains against MAP K10 reference strain.

MAPAC hybridisations comparing each vaccine strain against a MAPK10 reference control were made (in duplicate) and averaged values displayed as scatterplots (Figure 1a and Figure 1b). Significant loss of signals in contiguous genes representative of large variable genomic island (vGI) deletions were identified in a 26.8 Kbp region of 316FNOR1960 (vGI-19: MAP3714-MAP3735c; Table 1) and a 32.8 Kbp region in both IIUK2000 and 2eUK2000 (vGI-20: MAP1694-MAP1727; Table 2). Two fold increases in signals were also seen in contiguous genes within a 24.9 Kbp region of IIUK2000 (vGI-21: MAP2705c-MAP2733c; Table 3), a 40.7 Kbp region of 316 F-NLD1978 (vGI-22: MAP1750-MAP1789, Table 4) and a 11.0 Kbp 316FUK2000 (vGI-1b: MAP0096c-MAP0104; Table 5).

**Table 1:** Deleted region (vGI-19) in 316FNOR1960

**Gene**	**Name**	**Putative function**	**Process**
MAP3714	fadD2	LuxE like Acyl-coA synthase	Fatty Acid Metabolism
MAP3715		Unknown	
MAP3716c	fadE6	Acyl-CoA dehydrogenase	Fatty Acid Metabolism
MAP3717c		Dipeptidyl aminopeptidase	
MAP3718c		Transcriptional regulator	
MAP3719		Glyoxalase	Anti-host killing factor
MAP3720		Unknown	
*MAP3721		polyketide synthase associated protein PapA2	Cell Adhesion
*MAP3722		Unknown	
*MAP3723c		Transcriptional regulator	
*MAP3724		Unknown	
*MAP3725		PPE-1	Signalling
*MAP3726	fepD	ABC-type cobalamin/Fe3+ siderophore transporter	Transporter
*MAP3727	fepC	ABC-type cobalamin/Fe3+−siderophore transporter	Transporter
*MAP3728	fepB	ABC-type cobalamin/Fe3+−siderophore transporter	Transporter
*MAP3729	tauD	Taurine catabolism dioxygenase	Taurine resistance
*MAP3730	tehB	O-methyltransferase	Tellurite resistance
*MAP3731c		ABC non-ribosomal derived peptide transporter	Transporter
*MAP3732c		ABC non-ribosomal derived peptide transporter	Transporter
*MAP3733c		ABC non-ribosomal derived peptide transporter	Transporter
*MAP3734c		ABC non-ribosomal derived peptide transporter	Transporter
*MAP3735c		ABC non-ribosomal derived peptide transporter	Transporter

* = MAP specific genes.

**Table 2:** Deleted region (vGI-20) in vaccine strains IIUK2000 and 2eUK2000

**Gene**	**Name**	**Putative function**	**Process**
MAP1694	papA2	Polyketide synthase	Cell Adhesion
MAP1695c		Transcriptional regulator	
MAP1696c	hsp18_1	Heat Shock Protein	Stress protein
MAP1697		Transcriptional regulator	
MAP1698c	hsp18_2	Heat Shock Protein	Stress protein
MAP1699	thil	Thiamine biosynthesis	
MAP1700c		β-lactamase	
MAP1701c		Biotin carboxylase subunit	
MAP1702c		Thioredoxin	
MAP1703c		Unknown	
MAP1704c		Glyoxalase	Anti-host killing factor
MAP1705c		Transcriptional regulator	
MAP1706		Chitinase-like protein	
MAP1707		3-ketoacyl-(acp) reductase	Fatty Acid Metabolism
MAP1708		Phosphohydrolase-like protein	
MAP1709	fadD11_2	LuxE like Acyl-protein synthetase	Fatty Acid Metabolism
MAP1710		3-oxoadipate enol-lactonase	Fatty Acid Metabolism
MAP1711c		Transcriptional regulator	
MAP1712		CoA-transferase family III	
MAP1713	fadDE20_1	Acyl-CoA dehydrogenase	Fatty Acid Metabolism
MAP1714	fadA_2	Acyl-CoA dehydrogenase	Fatty Acid Metabolism
MAP1715	fadB_2	Acyl-CoA dehydrogenase	Fatty Acid Metabolism
MAP1716		Short chain dehydrogenase	Fatty Acid Metabolism
MAP1717		Unknown	
*MAP1718c		Low identity to MTB immunogen MPT63	Cell internalization factor
*MAP1719c		Transcriptional regulator	
*MAP1720		Nucleoside-diphosphate-sugar epimerases	Cell envelope biosynthesis
*MAP1721c		Transcriptional regulator	
*MAP1722		IS*900*	
*MAP1723		NADP oxidoreductase coenzyme F420-dependent	
*MAP1724c		Cytochrome b N	
*MAP1725c		Catalase	Anti-host killing factor
*MAP1726c		Transcriptional regulator	
*MAP1727		Esterase_lipase	Fatty Acid Metabolism

* = MAP specific genes.

**Table 3:** Tandem duplicated region (vGI-21) in vaccine strain IIUK2000

**Gene**	**Name**	**Putative function**	**Process**
MAP2705c		Ketosteroid isomerase	
MAP2706c		N-acylglucosamine 2-epimerase like	
MAP2707c		Signal protein	
MAP2708c		Glutamine amido transferase	Amino acid-Lipid biosynthesis
MAP2709c	tesB2	Acyl-CoA thioesterase II	Amino acid-Lipid biosynthesis
MAP2710c	pdxS	Pyridoxal 5’-phosphate synthase	Amino acid-Lipid biosynthesis
MAP2711c		Nudix Hydrolase 26	
MAP2712c		Phosphatidylinositol alpha-mannosyltransferase	
MAP2713c		Lauroyl acyltransferase	Lipid biosynthesis
MAP2714c	pgsA	CDP-alcohol phosphatidyltransferase	Lipid biosynthesis
MAP2715c		Diadenosine tetraphosphate	
MAP2716c	thrS	threonyl-tRNA synthetase	
MAP2717		Na+/H+ antiporter	
MAP2718c		N-alpha-acyl-glutamine aminoacylase	Amino acid biosynthesis
MAP2719c		Acyl-CoA thioesterase	Amino acid biosynthesis
MAP2720c		Aldo-keto reductase	
MAP2721		Unknown	
MAP2722c		2-Nitropropane dioxygenase	
MAP2723c		Unknown	
MAP2724c		Unknown	
MAP2725c		Transcriptional regulator	
MAP2726c	fdxA	Ferredoxin	Fe scavenging
MAP2727		S-adenosylmethionine-dependent methyltransferase	
MAP2728		Transcriptional regulator	
MAP2929c	pcaB	Lyase I like	Benzoate metabolism
MAP2730c	pcaG	Protocatechuate 3,4-dioxygenase	Benzoate metabolism
MAP2731c	pcaH	Protocatechuate 3,4-dioxygenase	Benzoate metabolism
MAP2732c		Hemerythrin oxygen binding like protein	
MAP2733c		Rieske (2Fe-2S) protein	

**Table 4:** Tandem duplicated region (vGI-22) present in vaccine strain 316FNLD1978

**Gene**	**Name**	**Putative function**	**Process**
MAP1750		Unknown	
MAP1751	wcaG	NAD-dependant epimerase/dehydratase	Cell envelope biosynthesis
MAP1752c		Aldo-keto reductase	
MAP1753		Acyltransferase	
MAP1754c	uspA	Adenine nucleotide alpha hydrolase	Stress Protein
MAP1755c	nirC	Formate/nitrite transporter	
MAP1756c	rshA	Anti-sigma factor	
MAP1757c		Sigma-70 factor	
MAP1758c	nrtC	Acyl-CoA dehydrogenase	
MAP1759c	osmC	Osmotic shock-like protein	
MAP1760c		tat translocated iron dependent peroxidise	
MAP1761c		Peptidase like lipoprotein	
MAP1762c		FTR1; high affinity iron transporter	
MAP1763c		Unknown	
MAP1764		Unknown	
MAP1765c	rssA	Patatin like esterase	Fatty acid Metabolism
MAP1766c		Cellobiose ABC transporter	
MAP1767c		ABC transporter	
MAP1768c		ABC transporter	
MAP1769c		Transcriptional regulator	
MAP1770c		Sigma-70 factor	
MAP1771c		IS*900*	
MAP1772		Unknown	
MAP1773c		Nuclear transport factor 2	Steroid metabolism
MAP1774c	pncA	Isochorismatase	Nicotinamide metabolism
MAP1775	doxX	Unknown	
MAP1776c		Nucleotide phosphokinase-like	
MAP1777c		Unknown	
MAP1778c	aroK	Shikimate kinase	
MAP1779c		Phosphotransferase	
MAP1780c	lipT	Esterase_lipase	Fatty acid Metabolism
MAP1781	lppI	Lipoprotein	
MAP1782c		p450 cytochrome	
MAP1783		Transcriptional regulator	
MAP1784c		Lipoprotein	
MAP1785		IS*900*	
MAP1786c		Dihydropicolinate reductase-like	
MAP1787c		Short chain dehydrogenase	
MAP1788		Transcriptional regulator	
MAP1789		Unknown	

**Table 5:** Duplicated region (vGI-1b) in vaccine strain 316FUK2000

**Gene**	**Name**	**Putative function**	**Process**
MAP0095c	nuoI_1	NADH dehydrogenase subunit I	
MAP0096c		Membrane fatty acid desaturase	Fatty acid metabolism
MAP0097c		Membrane fatty acid desaturase	Fatty acid metabolism
MAP0098c		Transcription regulator	expression regulator
MAP0099	csbD	Stress protein	stress response
MAP0100	gcs2	Glutamate-cysteine ligase	glutathione biosynthesis
MAP0101	gcs2	Glutamate-cysteine ligase	glutathione biosynthesis
MAP0102		Unknown	
MAP0103c		Unknown	
MAP0104	IS1311	Transposase	DNA mutator transposon

Signal ratios from genes in multiple copies in the genome such as insertion elements are more difficult to interpret as changes in signal are related to copy number. Nevertheless IS*900* signal ratios were significantly reduced in IIUK2000, 2eUK2000, 316FNOR1960, 316v, 316FUK2000 and elevated in 316FNLD1978 and 316FNLD2008. Increases in signals for IS*1652*-like elements were also seen in IIUK2000, 2eUK2000, 316FUK2000, 316FNLD1978 and 316FNLD2008 and decreases for this element in 316FNOR1960. IS*256*-like elements were more abundant in 2eUK2000 and also raised in 316v and 316FUK2000 but decreased in 316FNOR1960 relative to MAPK10. IS*1311*-like elements were increased in 316v and decreased in 316FNOR1960. One of these elements (MAP0104) borders the previously described vGI-1b region which is bounded by short inverted repeat sequences and duplicated in MAP type III strains [26].

The tandem duplication vGI-21 was confirmed using primer set MAP2705.seq2 and MAP2733.R (Table 6) orientated to amplify only the end of one tandem repeat and the beginning of the other. This produced an amplicon of 651 bp only with vaccine strain IIUK2000 and IIUK2001 (Additional File 1). Sequencing of this amplicon showed the duplication event to occur relative to the reference strain K10 genome (GenBank accession AE016958) at position 3066285 within the first copy of MAP2733c (truncated at aa251) followed by a unique region of 16 bp (CCAGCTCGGCCACCCG) and then a second copy of MAP2704 (truncated at aa138) followed by the vGI-21 duplication thus comprising a tandem duplication of 24,971 bp spanning 29 ORFs (MAP2705c-MAP2733c). PCR using primer pairs specific for this tandem duplication MAP2705.seq2 and MAP2733.R was negative for all other strains included in this study.

**Table 6:** PCR primer sets used in this study

**Gene set**	**Sequence**
vGI-1b: (internal gene primer sets)	
MAP0101.F	GGTTACCGACTTGGTCCAGA
MAP0101.R	CCCGTCAGATCCATTACGAC
MAP0103.F2	GAGCGCCAACCTATCTCAAC
MAP0103.R	TGGTTTGGAGATCCCTGAAG
vGI-19 (internal gene primer set)	
MAP3733c.F	TTCATGTTGTTCCTCACCCG
MAP3733c.R	CATCGCGAGCGTAGCCGCTG
vGI-20 (internal gene primer set)	
MAP1723.F	GGAGTATGGAACCATCGCTG
MAP1723.R	TGATGTAACGGGCGTGCAAG
vGI-21 (specific for tandem duplicated region in IIUK2000/1)	
MAP2705.SEQ2	CGATGAAGGCCTACTGGTC
MAP2733.R	TCAACTGGCTCCTCCTTTTG
vGI-22 (specific for tandem duplicated region in 316FNLD1978)	
MAP1789.F	TTTGGCACTACAACGAGCTG
MAP1750.R	GATCGAGAAGAGGGGAGTCA
MAP2114c (single copy gene for control PCR)	
MAP2114c.F	TGGAAGCTACCCAAATCACC
MAP2114c.R	GAGAAGTAGGCCGCGTAGTC
IS*900*	
AV-1	ATGTGGTTGCTGTGTTGGATGG
AV-2	CCGCCGCAATCAACTCCAG
pre16SrRNA	
pre16SrRNA.R	GCGCAGCGAGGTGAATTT
pre16SrRNA.F	TTTGGCCATACCTAGCACTCC
Tellurite (tehB)	
MAP3730.F	GGTTTGACGACAGAGATGCG
MAP3730.R	GGCATGGTGTACGACAAGGA
MIRU3 (MAP3981-2)	
MIRU3.F	ACATTCACCCTGTCCATTCC
MIRU3.R	CCTCCTTACGGAGCAGGAA
IS*900*(MAP0034)	
MAP0033c.F	AGCTGCCGGGAGTTGATCT
MAP0035.R	TCACGGCTCACCGCACGCT
IS*900*(MAP0159c)	
MAP0158.F	CCCGTGACAAGGCCGAAGA
MAP0160.R	CGTTTTGCACCTCGATGGCC
IS*900*(MAP0967)	
MAP0966c-MAP0967.F	CGTGACGAACGACATGTGTT
MAP0967-MAP0968.R	GCTCGAGACATTTAGCCCAC
IS*900*(MAP1033)	
MAP1032c.F	GTAGAACCCGACCAGCAGC
MAP1034c.R	GACCTGGCACCTGTATCCC
IS*900*(MAP1722)	
MAP1720-MAP1721c.F	CTAAGGTGGCCAGCGTTTCT
MAP1723.R	GGTTGGAGACAACCTCGTTC
IS*900*(MAP1771c)	
MAP1770c.F	ACAATTCGGCGATCGTCTCG
MAP1772.R	CGCGGACAGACACAGGTAGG
IS*900*(MAP1785)	
MAP1784c.F	GTCGACCATCGCTCTTCCCT
MAP1786c.R	ATCGGTGTCGAGGACATTCC
IS*900*(MAP1793c)	
MAP1792.F	CAATGGATGGTCGTCACCTG
MAP1794.R	CGGCCCGCTTGATCCATTTG
IS*900*(MAP2034c)	
MAP2033-MAP2034c.F	CCGCAAGTAGTGCACTATGG
MAP2035.R	TATTCGGGGTTGTTCAGGGA
IS*900*(MAP2108)	
MAP2105.F	CAGGCACGGAACACAGTTCG
MAP2109c.R	TGTTCGGCTACGGCATACTG
IS*900*(MAP2157)	
MAP2156.F	CTGCAAACACAGCCCAATC
MAP2158.R	CAACTTCGGCAAGTTCACC
IS*900*(MAP2203c)	
MAP2202c.F	TCCCGGTAGAAGATCATGTG
MAP2204c.R	GACAATCTGCCGTCGTATCA
IS*900*(MAP2444c)	
MAP2443.F	AACCTTGACCCACACCTTCC
MAP2445.R	TGAGCTCGCCGGCGAAATA
IS*900*(MAP2577)	
MAP2567c.F	CGTTCTGGGCATCCATCGACG
MAP2578.R	TCACGGCGGTCAGGTTACTTC
IS*900*(MAP3480)	
MAP3479c.F	GTTGAACTTTCCGACCAACC
MAP3480-3481.R	GGTTAGCGGGAGAAAAGCTC
IS*900*(MAP4066)	
MAP4065.F	TGGGCCTGAGGTCAGAACCA
MAP4067c.R	GAAGACCACCTCTACCTCAC
IS*900*(MAP4281)	
MAP4280.F	GCTGACCGAGAAGGGCTAC
MAP4282.R	CGTAAGTGACTGGCTCATGG

MAP gene annotation corresponds to GenBank AE016958.

vGI-22 was confirmed as a tandem duplication using primer set MAP1789.F and MAP1750.R (Table 6) which produced an amplicon of 2967 bp with vaccine strain 316 F-NLD1978 only. Sequencing of this region showed the duplication event to comprise 40,744 bp spanning 41 ORFs (MAP1749-MAP1789). The duplication site occurs at Genbank accession AE016958 position 1952589 within the first copy of MAP1790 (truncated at aa173) followed by an overlap region of 3 bp (GGG) and then the second copy of MAP1748c (truncated at aa143) followed by the vGI-22 duplication. PCR with primer pairs specific for this tandem duplication (MAP1789.F, MAP1750.R) was negative for all other strains included in this study.

Several attempts to localise the vGI-1b duplication using outward facing primers around the region in a similar manner to vGI-21 and vGI-22 were unsuccessful and this region may not be present in 316UK2000 as a tandem duplication. qPCR was performed using both MAP0101 and MAP0103 specific primer pairs located inside vGI-1b and the relative copy number of each compared against a single copy genome target control represented by primer pairs to MAP2114c. Both MAP0101 and MAP0103 pairs showed a doubling of signal strengths relative to MAP2114c in strain 316FUK2000 whilst all other strains included in this study showed signal consistent with single copies of both these genes (Table 7).

**Table 7:** **MIRU, vGI, Tellurite MIC and IS*900*/ MAP0101 / MAP0103 genome copy numbers**

**Strain**	**MIRU3**	vGIs*	**Tellurite MIC**	**Copy number relative to MAP2114c**	
	**bp (copy no)**		**μg/ml**	**IS*900***	**MAP*0101*/ MAP0*103***
316FNLD2008	244 (1)		128	17	1
316FUK2001	244 (1)		nd	nd	nd
316FNLD1978	244 (1)	22^dp^	128	19	1
316FUK2000	297 (3)	1b^dp^	128	16	2
316FNEO4/81	297 (3)		nd	17	1
316FNEO8/81	297 (3)		nd	17	1
316FNEO68451-2	297 (3)		nd	17	1
316FNEO69341	297 (3)		nd	17	1
316v	297 (3)		nd	16	1
316FCYP1966	297 (3)		> 512	16	1
316FNOR1960	297 (3)	19^dl^	64	16	1
IIUK2000	297 (3)	20^dl^ & 21^dp^	8	13	1
2eUK2000	297 (3)	20^dl^	8	13	1
MAPK10	297 (3)		128	17	1
CAM87	297 (3)		256	17	1

nd = not done.

* vGI status for vGI-1b, vGI-19 - vGI-22 either duplicated (dp) or deleted (dl), else no entry designates present as a single copy region.

### IS*900* insertion site analysis

To determine which IS*900* sites were absent relative to the K10 reference genome, PCR primers were designed to specifically amplify each of the known 17 IS*900* loci (Table 6). These were used to confirm the insertion of IS*900* into each locus in the reference strain K10 and were also all positive in all 316 F strains and a caprine isolate CAM87. Both vaccine strains IIUK2000 and 2eUK2000 were missing IS*900*(MAP1722) whereas IS*900*(MAP1033) was also missing from vaccine strain 2eUK2000 but present in all other strains including vaccine strain IIUK2000. Comparative qPCR of IS*900* copy number relative to MAP2114c, demonstrated a range of IS*900* copies in vaccine strains that corresponded to the trend in hybridisation signals observed in MAPAC scatterplots (Figure 1a &1b). The ratio of copy number however was surprisingly higher than predicted, with vaccine strains IIUK2000 and 2eUK2000 having only 13 copies whilst MAPK10 and 316 F strains gave signals correspondent with 16–19 relative IS*900* copy numbers (Table 7).

### Functional analysis of tellurite resistance

One MAP specific gene predicted to be deleted in vGI-19 was MAP3730 (Table 1), a S-adenosylmethionine-dependent methyltransferase with homologues to tellurite resistance genes (tehB) involved in bacterial virulence and persistence [27,28]. The functionality of this gene in mycobacteria has not previously been investigated. Using a solid culture plate assay we compared tellurite resistance (MIC) of MAP strains with and without the vGI-19 deletion (Table 7). This demonstrated a wide MIC range (8 - >512 μg/ml) between strains, with significant reductions associated with vGI-19 (316FNOR1960) deletion over full genome complement strains. Of note however was the very low level of tellurite resistance (8 μg/ml) found in strains containing the vGI-20 (IIUK2000 & 2eUK2000) deletion.

### Assessment of virulence using a mouse model

The virulence of vaccine strains 316FUK2001, IIUK2001 and 2eUK2001 was compared with wild type strain JD87/107 in a mouse model. Ten mice from each of five groups (four inoculated with the different MAP strains and a negative control group inoculated with PBS) were killed at 4, 8 and 12 weeks post inoculation. Body, spleen and liver weights were recorded. Samples of the liver were taken for bacteriological culture and histopathology. Mean bodyweights increased with age, but no statistically significant difference was observed in mean body weight between any of the vaccine strains and the control wild type strain at any of the time points (p=0.11). There was strong evidence of a difference in pattern over time between the mean rank spleen weight expressed as a percentage of body weight of the mice inoculated with the different MAP strains relative to those receiving PBS, where those inoculated with MAP had increasingly higher mean rank percentage weights (P=0.006). Differences between the MAP strains were not formally statistically significant (p=0.06) although the control virulent strain JD87/107 showed an increase in mean rank spleen weight percentage between weeks 4 and 8, and 316FUK2001 had an increase between weeks 8 and 12. There was no statistical evidence for differences in the mean levels of liver weight expressed as a percentage of body weight either for different strains or over time for any of the MAP strains (p = 0.2). However, there was some evidence of a difference between the means for the MAP strains and the lower mean weights associated with PBS (p = 0.018).

**Figure 2:**
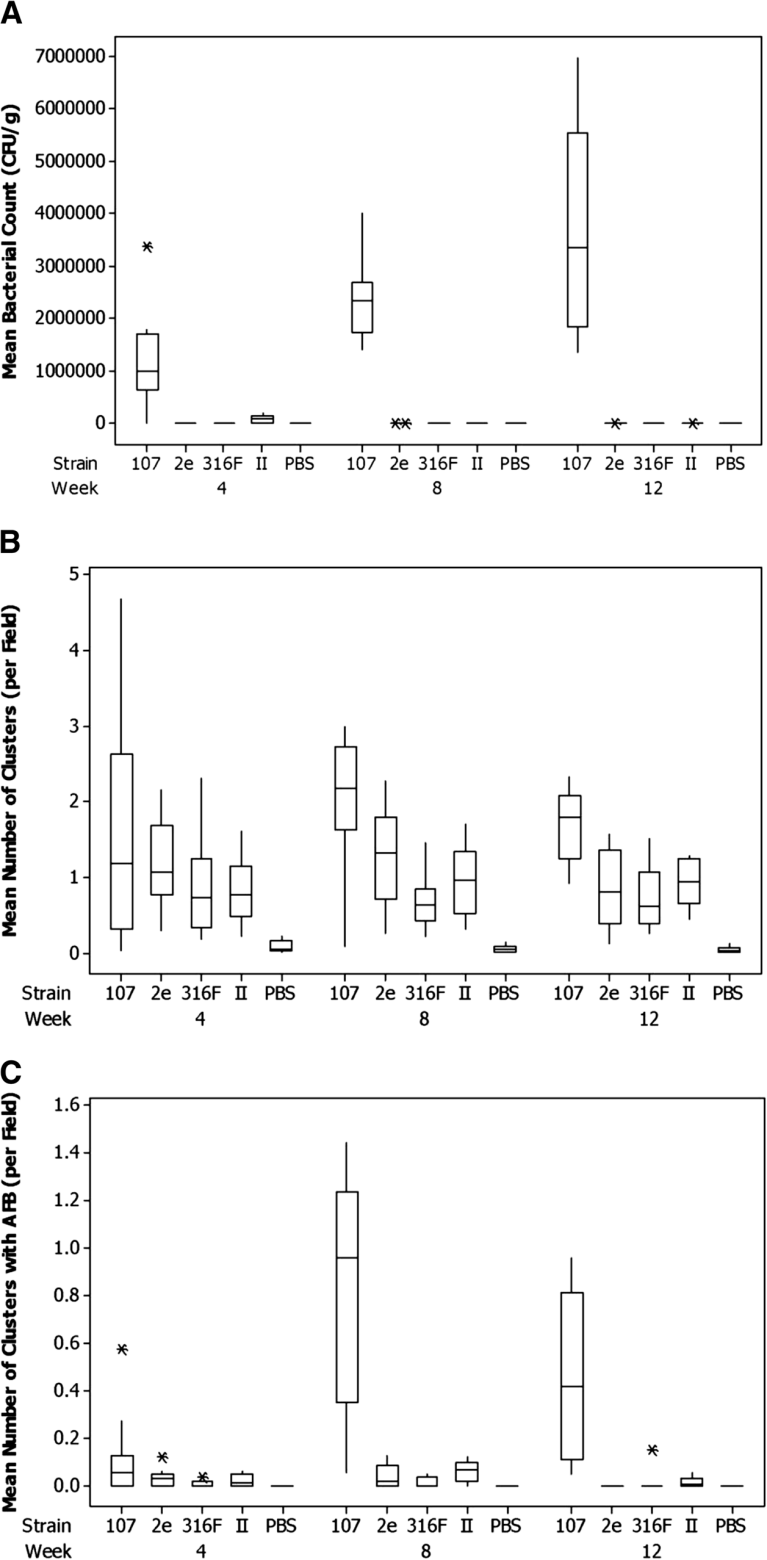
**Virulence assessment of vaccine (2eUK2001, 316FUK2001, IIUK2001) and wild type (JD87/107) MAP strains in a mouse model. A**. Quartile-Based Box and Whisker plots of bacterial load (CFU/g) in the liver at 4, 8 and 12 weeks post-infection. **B**. Quartile-Based Box and Whisker plots of mean ranked density of leucocyte clusters in the liver at 4, 8 and 12 weeks post-infection. **C**. Quartile-Based Box and Whisker plots of mean ranked density of leucocyte clusters with AFB in the liver at 4, 8 and 12 weeks post-infection. * indicates an unusually large or small observation (outlier). Values beyond the whiskers are outliers. The top of the box is the third quartile −75% of the data values are less than or equal to this value. The bottom of the box is the first quartile −25% of the data values are less than or equal to this value. The median is shown within the box. The whiskers extend to the highest and lowest data values which have not been identified as outliers.

MAP was recovered from the liver tissue of mice four weeks post inoculation in all groups except the control group inoculated with PBS. By 12 weeks post infection, MAP was recovered from the tissues of only one mouse inoculated with vaccine strain 2eUK2001 (mean count 46 cfu/g), from 6 mice inoculated with IIUK2001 (mean counts between 46 and 315 cfu/g) and from all the mice inoculated with the virulent JD87/107 strain (mean counts 1.4-7 × 10^6^ cfu/g) suggesting attenuation of each of the vaccine strains (Figure 2a). Mean rank counts increase over time for the JD87/107 strain, while dropping for all the other MAP strains, this being most rapid for the 2eUK2001 strain but ultimately most notable for strain 316FUK2001. Statistical assessment of the effect of the strain by time interaction on the mean rank count indicate that differences exist in the abilities of the MAP vaccine strains to survive or persist in mice (p=0.02).BMC1010

Infections of the liver result in multifocal hepatitis characterised by clusters of inflammatory cells. To measure the effect of the experimental infection with MAP, the densities of mononuclear leucocyte clusters were counted. Small numbers of leucocyte clusters (< 0.22 clusters per field) were observed in liver samples from mice inoculated with PBS, though no acid fast bacilli (AFB) were detected in any of these samples. Large differences in the mean ranked density of leucocyte clusters between strains were identified (p<0.001) with the wild type strain JD87/107 having the highest mean ranked densities of clusters (Figure 2b). Strain 2eUK2001 showed evidence of higher mean rank densities than the 316FUK2001 and IIUK2001 strains (p = 0.03). The ranked density of leucocyte clusters with AFB showed highly statistically significant differences between the means of MAP strains (p<0.001), with the JD87/107 strain consistently showing higher mean densities, with this effect being more pronounced from 8 weeks post infection (Figure 2c). The vaccine strains all tended to exhibit increasingly lower mean ranked densities over the lifetime of the experiment (p=0.002), with consistent patterns of differences between strains (p=0.008): strain IIUK2001 showed the largest mean rank densities, strain 316FUK2001 the lowest, with 2eUK2001 intermediate. The histopathology results show that all strains elicited a similar inflammation at 4 weeks. Only thereafter some differences between the inflammatory responses to the strains became apparent. In addition, the analysis of mean bacterial counts and AFB positive clusters showed the reduced ability of the vaccine strains to survive and persist within mice. Overall, these results provide proof of attenuation of the vaccine strains with respect to a wild type MAP strain.

## Discussion

In this study, we examined genomic and phenotypic characteristics of a panel of MAP vaccine strains obtained from several laboratories around the world including both low and high passage examples of the 316 F lineage. Using a mouse model, we assessed the virulence ofrepresentative clades of three vaccine strains (2e, II, 316 F) with respect to a virulent MAP clinical isolate. The vaccine strains were clearly attenuated with regard to their ability to survive and persist in the mice as evidenced from the reduced numbers of MAP recovered and reduced numbers of leucocyte clusters containing AFB in the livers. This supports previous studies showing decreased persistence of the same 316 F and 2e strains in calves after 8 months [29] and illustrates the utility of the C57BL/6 mouse model for virulence studies.

Using a pan-genomic MAP/MAH microarray we demonstrated that the genomes of all but one of the 316 F strains in the test panel contain the same full genome complement as the reference virulent bovine MAP type II strain MAPK10. One 316 F strain obtained from Norway (316FNOR1960) contained a single deleted region (vGI-19) spanning 21 ORF’s (including 10 MAP specific genes). Two strains not of the 316 F lineage (2eUK2000 and IIUK2000) contained a different deleted region (vGI-20), identical in both strains, spanning 34 ORF’s (including 10 MAP specific genes). Specific PCR for MAP1723 (Table 6), located within the vGI-20, on 2eUK2001 and IIUK2001 were negative confirming that the vGI-20 region was not present in these strains (Additional File 1). The inclusion of MAP-specific genes in these deleted regions is an important observation as these genes could provide the basis for differentiating infected from vaccinated animals (DIVA). Indeed, the deleted region vGI-19 contains part of the 38 kb pathogenicity island described by Stratmann *et al.* (2004) [30] which contains genes encoding a number of antigens with diagnostic potential [31]. Both deleted regions vGI-19 and vGI-20 contain genes potentially involved in virulence and pathogenesis (Table 1) and their deletion could therefore have a profound effect on the virulence of these strains. In this study we demonstrated using a mouse model that both vaccine strains 2eUK2001 and IIUK2001 were attenuated with respect to a wild type MAP strain. In addition, vaccine strain IIUK2000 and IIUK2001 were found to contain a large 41 ORF tandem duplication (vGI-21) which includes copies of benzoate and lipid metabolic pathways. Vaccine strain 2eUK2000 comes from the same stock as 2eUK2001 and was maintained at the VLA, UK for over 50 years on a mineral deficient medium (Watson Reid ‘A’ block) whilst vaccine strain IIUK2000 was not. We suggest that the vGI-21 duplication in vaccine strain IIUK2000 was selected by these differences in media and fixed into the genome to compensate *in vitro* for the deletion of lipid biosynthesis and carbon usage repertoires, removed by the vGI-20 deletion.

The large deletion vGI-19 present in vaccine strain 316FNOR1960 was not present in any of the other 316 F strains including an early low passage lineage (316FCYP1966) and a more recent isolate (316 F2001) shown to be significantly attenuated in our virulence studies. Notably, part of vGI-19 is also present in the same gene order within the related MAH104 genome (GenBank reference CP000479). Together these suggest that any ancestral precursor and therefore the original 316 F strain would be unlikely to be missing vGI-19. We hypothesise that the vGI-19 deletion appeared in the 316FNOR1960 strain some point after its acquisition and transfer to Norway in 1960 from the VLA, UK. This strain is recorded as having been maintained, uniquely on Dubos medium with added pyruvate [15] and we hypothesise that this medium was at some point selective. This is supported by the vGI-19 deletion in this strain including gene homologues of glyoxylate enzymes associated with pyruvate metabolism [32]. This strain previously has been used successfully as a live vaccine suggesting that it is attenuated. The knockout of the glyoxylate shunt could significantly affect the strain’s ability to control anaerobic respiration [33] and intracellular persistence [34], which may indicate that attenuation in this strain may be related to this loss. A 2002 isolate of this strain was shown to be highly immunogenic in goats [35] and forms part of a 316 F/2e live commercial vaccine product (Paratuberkulose-vaksine, National Veterinaria Institute, Oslo, Norway) however there is no evidence that vGI-19 was deleted when the strain was used, albeit very effectively, on goats in the 1960-70’s [15].

We tested the potential impact provided by deletion of the putative tellurite resistance gene (tehB) included in vGI-19 on 316FNOR1960 phenotype. Tellurite is highly toxic to bacteria due to its action on DNA synthesis. It is an important mechanism by which animals combat intracellular microorganisms [27] and was used in early studies as a tuberculosis/leprosy therapeutic [36]. Bacterial resistance to tellurite is inducible, is associated with virulence [28] and is linked to catalases which are required to process the superoxide anions generated as a result of bacterial metabolic mechanisms used to inactivate tellurite. We show a significantly increased sensitivity to tellurite in 316FNOR1960 whilst other 316 F strains either matched or exceeded the resistance of the two wildtype strains tested (K10:bovine, CAM87:caprine). Interestingly the strains most sensitive to tellurite were IIUK2000 and 2eUK2000 which lack the tehB gene. The metabolism of tellurite generates high reactive oxygen species which subsequently need to be de-toxified by catalase [37]. Significantly the vGI-20 deletion in these strains includes loss of the catalase gene homologue MAP1725c. Both vaccine deletion regions thus involve alterations in metabolic pathways associated with deactivation of high reactive oxygen species toxicity, which suggests this may be an important mechanism underlying attenuation in these strains.

Several of the other vaccine strains tested are also reported to have been maintained on markedly different media which may have similarly promoted or selected for genomic and phenotypic diversities. 316FNLD1978, available as a heat killed vaccination for dairy cattle since 1985 [38], was found to contain a large tandem duplication (vGI-22) unique to this strain. It is notable that this isolate was selectively subcultured on potato starch medium to enhance its growth (P. Willemsen personal communication) and now grows with difficulty on other media. It is tempting to speculate that the acquisition of extra copies of 14 ORFs including cell wall, fatty acid biosynthesis genes and two extra copies of IS*900* are a direct result of the selective process performed on this strain.

We demonstrated in this study that vaccine strain 316FUK2001 was clearly attenuated with respect to wild type MAP strain JD87/107. The vGI-19 deletion found in 316FNOR1960 and the vGI-20 deletion found in 2eUK2000 and IIUK2000 were not detected by PCR in this strain suggesting that attenuation in this strain is due to different genetic polymorphisms. A duplicated region (vGI-1b) was detected in vaccine strain 316FUK2000, which may possibly have arisen as an adaptation to growth on liquid Watson Reid media.

Insertion sequences bordering specific genomic regions have previously been associated with variably tandem duplicated [39] and possibly large genomic inversions [40] and contribute highly to the degree of plasticity in the MAP genome. Variations in copy number of insertion elements including IS*900*, IS*1311*, IS*256* and IS*1652*-like elements were seen between vaccine strains and virulent isolates. An IS*1311* was found immediately bordering the vGI-1b region duplicated in 316 F-UK2000 but not other 316 F strains. Similar genomic variations including vGI-1b have been observed in virulent MAP strains [26]. IS*900*, a definitive element of MAP found in all clinical and vaccine strains, was also shown to be present in a variety of copy numbers. This work used comparative ratios of qPCR signals to estimate the average number of IS*900* copies per cell per culture relative to two single copy MAP genes using an assumption determined from a MAP assembled genome sequence that MAPK10 would contain 17 copies. Our results confirm previous studies showing the vaccine strain 316v used in Australia for ELISA testing [41] contains one less genomic copy of IS*900*[42] than most other 316 F strains [25]. Vaccine strain 316FNLD1978 exhibited higher gene signal ratios consistent with the two extra copies of IS*900* copies inside the duplication of vGI-22. Vaccine strains IIUK2000 and 2eUK2000 contained lower signal ratios consistent with loss of an IS*900* copy inside the deletion region vGI-20. Consistently however the calculated IS*900* copy number in these strains was much lower than expected using the ratio method. Using site specific PCR we confirmed 16 IS*900* filled insertion sites in the genomes of these strains whereas the ratio method, using MAPK10 as a standard, predicted only 13 copies. The reason could be technical, perhaps involving incomplete bacterial lysis of these unusual strains, however IS*900* is known to replicate in episomal minicircles [43] and when all consensus insertion sites are filled they may exist as extra genomic components awaiting transposition. If this is indeed the case, virulent MAP strains would have the capacity to contain more than the predicted 17 IS*900* copies per cell. This could be an important factor in studies relying on qPCR to determine accurate estimates of MAP load [44].

MIRU3 is a short tandem repeat sequence located within the sensX3-regX3 two component signalling system that controls carbon source usage and mechanisms reducing damaging reactive oxygen species generated by aerobic metabolism [45]. The attenuated BCG vaccine characteristically contains a low MIRU3 tandem repeat copy number which has been suggested to be involved in the control of sensX3-regX3 expression [46]. In this study 316 F strains (316FNLD1978, 316FUK2001, 316FNLD2008) had low MIRU3 copy numbers whilst others, mostly originating from older culture stocks, were larger. This may represent a potential for genomic drift within recent 316 F lineages and could be used as a marker for the most current 316 F VLA, UK strain but may also be suggestive that loss of intracellular persistence in full genome complement 316 F strains may be associated with reduced transcriptomic signalling capacity. It will be of interest therefore in future total genome sequencing studies to compare dysfunctional SNP variations within signalling features of 316 F strain genomes.

## Conclusions

This study has shown that significant genomic diversity exists between MAP vaccine strains and within the 316 F lineage. These include large deletions, duplications and changes in insertion sequence copies. These mutations were probably derived in a classical manner by selective subculture on laboratory media and in some cases have led to significant alterations of phenotype and attenuation. There were 25 MAP specific gene deletions identified of which at least one could be linked to phenotypic change that would disadvantage its persistence in the host and thus associates it with virulence. Furthermore, these MAP-specific gene deletions could provide the basis for a DIVA diagnostic for use with these vaccines. Overall, this work illustrates that MAP genome plasticity can be influenced by *in vitro* culture over long periods and a robust definition of vaccine strain genome lineage will be necessary in the future to guarantee consistency between studies.

## Methods

### Strains and culture media

MAP strains used in this work, their origins, sources and media used for propagation are described in Table 8.

**Table 8:** Details of MAP strains used in this study

**Name**	**Origin and source**	**Medium used for maintenance and propagation**
316FNOR 1960 (Vaccine strain)	Obtained from the VLA in 1960 and used in a vaccine trial in goats in Norway during the 1960s [15]. Maintained at the Norwegian Veterinary Institute, Oslo.	Selective Dubos medium [47] supplemented with mycobactin (2 μg/ml) and pyruvate (4 mg/ml)
316FCYP1966 (Vaccine strain)	Obtained from the VLA in 1966 as lyophilised aliquots and used to vaccinate goats in Cyprus during the 1960s [18]. Strain used in this study was recovered from an aliquot lyophilised on 04 January 1966 and resuscitated in 2009 with limited passage since.	7H9^*^
316FNLD1978 (Vaccine strain)	Obtained from the VLA in 1978 and used as a killed vaccine [38]. Maintained at the Central Veterinary Institute, Lelystad, Netherlands.	Potato starch medium (P.Willemsen personal communication)
316FNEO4/81 (Vaccine strain)	Neoparasec vaccine (Merial, France) subcultures from a stock [25] assumed to be derived from a 316 F Weybridge UK strain purchased in the 1980s.	7H9^*^ or 7H11^**^
316FNEO8/81 (Vaccine strain)		
316FNEO68451-2 (Vaccine strain)		
316FNEO69341 (Vaccine strain)		
316v	Australian strain derived from a variant labelled 316f around 1986 [48] which itself was obtained from a New Zealand source who obtained the strain in the early 1980s. Maintained at the University of Sydney, Sydney, Australia.	Mycobactin free medium but grew poorly (R Whittington, personal communication)
316FUK2000 (Vaccine strain)	Obtained from the VLA in 2000 and maintained at St George’s Hospital Medical School, London, UK, with limited passage.	Liquid Watson Reid^†^
316FUK2001 (Vaccine strain)	Obtained as a lyophilised stock from the VLA in 2001 and maintained at the Moredun Research Institute, Scotland, UK.	7H11^**^
316FNLD2008 (Vaccine strain)	Obtained from VLA in 2008 and maintained at the Central Veterinary Institute, Lelystad, Netherlands	HEYM
IIUK2000 (Vaccine strain)	Obtained from the VLA in 2000 and maintained at St George’s Hospital Medical School, London, UK.	Liquid Watson Reid^†^[14]
IIUK2001 (Vaccine strain)	Obtained as a lyophilised stock from the VLA in 2001 and maintained at the Moredun Research Institute, Scotland, UK.	7H11^**^
2eUK2000 (Vaccine strain)	Obtained from the VLA in 2000 and maintained at St George’s Hospital Medical School, London, UK.	Liquid Watson Reid ‘A’ Block^††^ medium
2eUK2001 (Vaccine strain)	Obtained as a lyophilised stock from the VLA in 2001 and maintained at the Moredun Research Institute, Scotland, UK.	7H11^**^
MAPK10 (Wild type strain)	Purchased from ATCC: BAA-968. Sequenced reference strain isolated from a cow in 1990.	7H9^*^ or 7H11^**^
CAM87 (Wild type strain)	MAP Type III strain isolated from a goat in 2005 [26] and maintained at the Universidad Complutense de Madrid, Madrid, Spain.	7H9^*^
JD87/107 (Wild type strain)	Isolated from a deer in 1987 and maintained at the Moredun Research Institute, Scotland, UK.	7H11^**^

^*^7H9: Middlebrooks 7H9 (Becton Dickinson, UK) supplemented with 2 mg/L Mycobactin J (Allied Monitor, USA), 0.5% glycerol, 10% oleic acid-albumin-dextrose-catalase (OADC) enrichment medium (Difco, UK), 25 mg/L amphotericin B, 35 mg/L naladixic acid and 35 mg/L vancomycin.

^**^7H11: Middlebrooks 7H11 (Becton Dickinson, UK) agar supplemented with 2 mg/L Mycobactin J (Allied Monitor, USA), 2.5% glycerol (v/v), 10% OADC (v/v) enrichment medium (Difco, UK), 20% (v/v) new born calf serum, 0.3 g/L asparagine, Mycobacteria Selectatabs (10 mg/L amphotericin B, 200,000 units/L polymixin B, 100 mg/L ticarcillin and 10 mg/L trimethoprim [MAST Laboratories Ltd, UK]).

^†^Liquid Watson Reid: Asparagine 5 g/l, Potassium dihydrogen phosphate 2 g/l, Magnesium suphate 1 g/l, Tri-ammonium citrate 2 g/l, Sodium chloride 2 g/l, D(+) Glucose 10 g/l, Glycerol 48 ml/l, Ammonium iron (III) citrate brown 0.075 g/l, 1.33mls of Supplement A: 2 g/l Zinc sulphate, 2 g/l Copper sulphate, 1 g/l Cobalt nitrate, 1.33mls of Supplement B; 50 g/l Calcium chloride.

^††^Liquid Watson Reid ‘A’ Block: as Watson Reid medium but without supplements A and B.

### DNA extraction

DNA was extracted for typing and arrays using a previously described protocol [26]. Briefly, 1x10^9^ cells of cultures grown on liquid Middlebrook 7H9 medium for up to 12 weeks were pelleted, washed once in 1x PBS, then resuspended in 650 μl mycobacterial lysis buffer (0.5 M EDTA –pH 8.0-, 5 M NaCl, 1 M TrisHCl, 10% SDS and 8.6 ml H_2_O). Colonies were emulsified by passing 10 times through a 25 G needle, and treated overnight at 37°C with 5 μl lysozyme (Stock 100 mg/ml: Sigma), 10 μl proteinase K (Stock 10 mg/ml: Sigma) and 4 μl lipase (Stock 121 mg/ml Sigma). Samples were ribolysed (Lysing Matrix B; Qbiogene) at 6,500 rpm for 50 sec, iced for 10 min then 400 μl of lysate added to 400 μl of Qiagen DNAeasy AL lysis buffer, mixed and applied to a DNAeasy column. 200 μl of 100% ethanol was added and columns centrifuged at 8,000 rpm for 1 min, washed in 500 μl Qiagen Lysis buffer 1 and 2, then eluted in 90 μl DNA/RNAse free H_2_O overnight on the column at 4°C.

### MIRU3 typing and IS*900* locus PCR

Five microlitres of MAP DNA extracted from test strains was amplified for MIRU [49] or IS*900*[40] as previously described using 2 μM primers MIRU3.F& MIRU3.R spanning the MAP3982-MAP3983 locus or with IS*900* locus specific primers designed to amplify across the complete IS*900* insertion from immediately adjacent loci (Table 6). All PCR reactions used 1x Expand reaction buffer containing 1.5 mM MgCl_2_, 10% DMSO, 100 μM dNTP and 1 unit Expand High Fidelity Taq polymerase (Roche). Cycling conditions were: 95°C: 3 min: 1 cycle; 94°C: 30 sec : 60°C: 30 sec : 72°C : 1 min : 35 cycles; 72°C : 5 min : 1 cycle. Confirmation of amplicon product size in bp was made on 1.8% agarose gels.

### MAPAC microarray hybridisation and analysis

DNA from the test strain and reference MAP K-10 strain were fluorescently labelled and hybridised to the microarray using protocols described previously [50]. Briefly, 1 μg of DNA was labelled by random priming with Klenow polymerase to incorporate either Cy3 or Cy5 dCTP (GE Healthcare) for the test strain and reference strain respectively. Equal amounts of the Cy3 and Cy5 labelled samples were co-purified through a Qiagen MinElute column (Qiagen), mixed with a formamide-based hybridisation solution (1×MES, 1 M NaCl, 20% formamide, 0.02 M EDTA, 1% Triton) and denatured at 95°C for 2 min. The labelled sample was loaded on to a prehybridised (3.5×SSC, 0.1% SDS, 10 mg/ml BSA) microarray under two 22×22 mm LifterSlips (Erie Scientific), sealed in a humidified hybridisation cassette (Corning) and hybridised overnight by immersion in a waterbath at 55°C for 16–20 h. Slides were washed once in 400 ml 1×SSC 0.06% SDS at 55°C for 2 min and twice in 400 ml 0.06×SSC for 2 min. Microarrays were scanned using an Affymetrix 428 scanner, and signal intensity data were extracted using BlueFuse for Microarrays v3.5 (BlueGnome). The intensity data was further post-processed using BlueFuse to exclude both controls and low confidence data (p<0.1) prior to normalisation by 2D Lowess (window size=20) and median centring. Further analysis of the normalised data was undertaken using BlueFuse, GeneSpring 7.3.1 (Agilent Technologies) and Eisen Cluster [51]. Fully annotated microarray data has been deposited in BμG@Sbase (accession number: E-BUGS-264; http://bugs.sgul.ac.uk/E-BUGS-264) and also ArrayExpress (accession number: E-BUGS-264).

### qPCR

Relative copy number of amplicons were generated from IS*900*, MAP0101 and MAP0103 primer sets and compared against absolute amplicon numbers obtained from a MAP2114c primer set (Table 6). Reactions comprised 2 μl genomic DNA sample, 12.5 μl Power SYBR green mastermix (Applied Biosystems, Cat 4368706), 2 pMoles appropriate primer pairs, made to 25 μl with RNAse free H2O. PCR cycling used 95°C:15mins (1 cycle); at 95°C:30secs, 58°C :1 min, 72°C:1 min (40 cycles) with data collection at 76°C (10secs) using a CFX96 qPCR cycler (BioRad, UK). Sample copy numbers were estimated from an averaged value of three qPCR’s on each sample using a dilution curve of a control stock total genomic DNA MAP K-10 preparation serially diluted 10 fold to contain between 1 × 10^2^-10^6^ genome copies.

### Tellurite MIC

Cultures of MAP strains were grown in conventional liquid media to exponential phase for 6 weeks then adjusted to 10^4^ cfu/ml using OD550. Aliquots (10 μl) were inoculated onto solid RAF medium in petri dishes containing serial dilution of potassium tellurite to final concentrations of 512, 256, 128, 64, 32, 16, 8, 4 or 0 μg/ml and incubated at 37°C. MIC’s were taken as the least tellurite concentration able to inhibit >90% growth, seen as black colonies, after 6 weeks of growth and 12 weeks growth for strain IIUK2000 which was slower to grow *in vitro*.

### Assessment of virulence using a mouse model

The virulence of vaccine strains 316FUK2001, IIUK2001 and 2eUK2001 was compared with wild type strain JD87/107 in a mouse model. 316FUK2001, IIUK2001 and 2eUK2001 were selected to represent the three different vaccine strains that have been used for control of JD over the years. JD87/107 was selected as the control strain as this was the virulent wild type strain that was used previously in our laboratory to optimise the mouse model and PBS was used as a negative control. C57BL/6 mice of approximately five weeks old and between 20 and 25 g in weight were purchased from Harlan UK, Shaws Farm, Blackthorn, Bicester, Oxon OX25 1TP. The mice were individually weighed and randomly assigned to five groups of 30. One negative control group was inoculated with 0.1 ml of sterile PBS. The remaining groups were inoculated intraperitoneally with 1.1 to 1.4 × 10^8^ organisms in 0.1 ml PBS of one of the MAP strains 2eUK2001, IIUK2001, 316FUK2001 and JD87/107. The inocula were prepared as previously described [52] and enumerated by performing a microscopic count. Ten mice from each group were killed at 4, 8 and 12 weeks post inoculation by exposure to a mixture of carbon dioxide and halothane gas followed by cervical dislocation. Each mouse was weighed and the body weight recorded. The spleens and livers were removed aseptically and weighed. The respective weights were expressed as a percentage of body weight for each mouse. Approximately 0.1 g of liver was removed for bacteriological culture and the remaining tissue fixed in 10% formal saline for histopathological examination.

Hepatic bacterial load was assessed by using serial dilutions of a 10% (wt/wt) liver homogenate in PBS. 0.1 ml of each dilution was inoculated onto 7H11 agar with supplements as detailed in Table 8 and incubated at 37°C for up to 16 weeks. Colony counts for each animal replicate were estimated by fitting a generalised linear model to the dilution assay counts assuming an overdispersed Poisson response and a logarithmic link function while fitting the logarithm of the dilution as an offset variable to the fixed mean. It was assumed that observations greater than 200 CFU per field could not be quantified accurately, and such observations were included in the likelihood as taking unknown values greater than this threshold.

The fixed liver samples were given a random code to assure that the samples were assessed blind by the pathologist. The samples were processed to paraffin wax and 5 μm sections prepared. The sections were stained with haematoxylin and eosin (H&E) and Ziehl-Neelsen (ZN) method . Using an Olympus BX50 microscope, the number of leucocyte clusters was counted in approximately 100 fields at ×200 magnification from each individual animal using an eyepiece graticule (Pyser-SGI Ltd, NE35-24 mm) and the counts normalised to 1 field. A leucocyte cluster was defined as an accumulation of more than 10 mononuclear leucocytes. The infectious load of each animal was assessed by counting leucocyte clusters containing AFB. Because the detection of AFB requires higher magnification (x400-600), the number of leucocyte clusters with AFB was assessed separately. Depending on the original leucocyte cluster density, up to sixty leucocyte clusters were assessed in detail and the proportion of leucocyte clusters containing AFB determined. Based on the leucocyte cluster counts and the proportion of leucocyte clusters containing AFB, the infectious load was expressed as the mean number of AFB positive leucocyte clusters per field.

All data were analysed by fitting a linear mixed model to either the data as specified above or to the ranks of these data, with this choice being made on the basis of the normality of residuals in the model fitted to the original data. The mixed model approach was preferred to traditional ANOVA to better allow for replicates missing at random from the sample. Strain and Week and interactions were fitted as fixed effects, animal replicate as a random residual effect. Statistical analysis was carried out using Genstat version 14 and using user-defined macros in Excel 2007. All statistical analysis and derivations of P values are provided in Additional File 2).

### Ethical considerations

All experimental procedures and management protocols were examined and approved by the Moredun Research Institute Experiments and Ethics Committee and conducted within the framework of the UK ‘Animals (Scientific Procedures) Act 1986’ administered by the Home Office of the UK government.

## Competing interests

The authors declare that they have no competing interests.

## Authors’ contributions

TB conceived of the study, carried out the molecular studies and data analyses and drafted the manuscript. AS contributed to conception and design and performed the histopathological examinations and data analysis of the mouse studies. JMS conceived of the study, participated in design, execution and analysis of the mouse studies. MG participated in the mouse studies. IJM and JS participated in the design, carried out statistical analyses of data from the mouse studies and contributed statistical text to the final manuscript. RL carried out the molecular studies and data analyses. KS conceived of the study, participated in the design, coordination, execution and analysis of the mouse studies and help draft the final manuscript. All authors read and approved the final manuscript.

## Supplementary Material

Additional file 1 **PCR amplification for vGI-19, vGI-20 and vGI-21 in 316FUK2001, 2eUK2001 and IIUK2001 strains.** Gels of specific PCR amplicons. Click here for file

Additional file 2 **Mouse Model Data File.** Tables and statistical analyses of virulence experiments in mice. (DOCX 86 kb) Click here for file
