# Supplementary material for: Genomic variations associated with attenuation in Mycobacterium avium subsp. paratuberculosis vaccine strains
Source: BMC Microbiol. 2013 Jan 22;13:11. doi: 10.1186/1471-2180-13-11 (PMC3599157; doi:10.1186/1471-2180-13-11)
Supplement: Additional file 1 — PCR amplification for vGI-19, vGI-20 and vGI-21 in 316FUK2001, 2eUK2001 and IIUK2001 strains. Gels of specific PCR amplicons. [file 1471-2180-13-11-S1.pptx]

## Slide 1
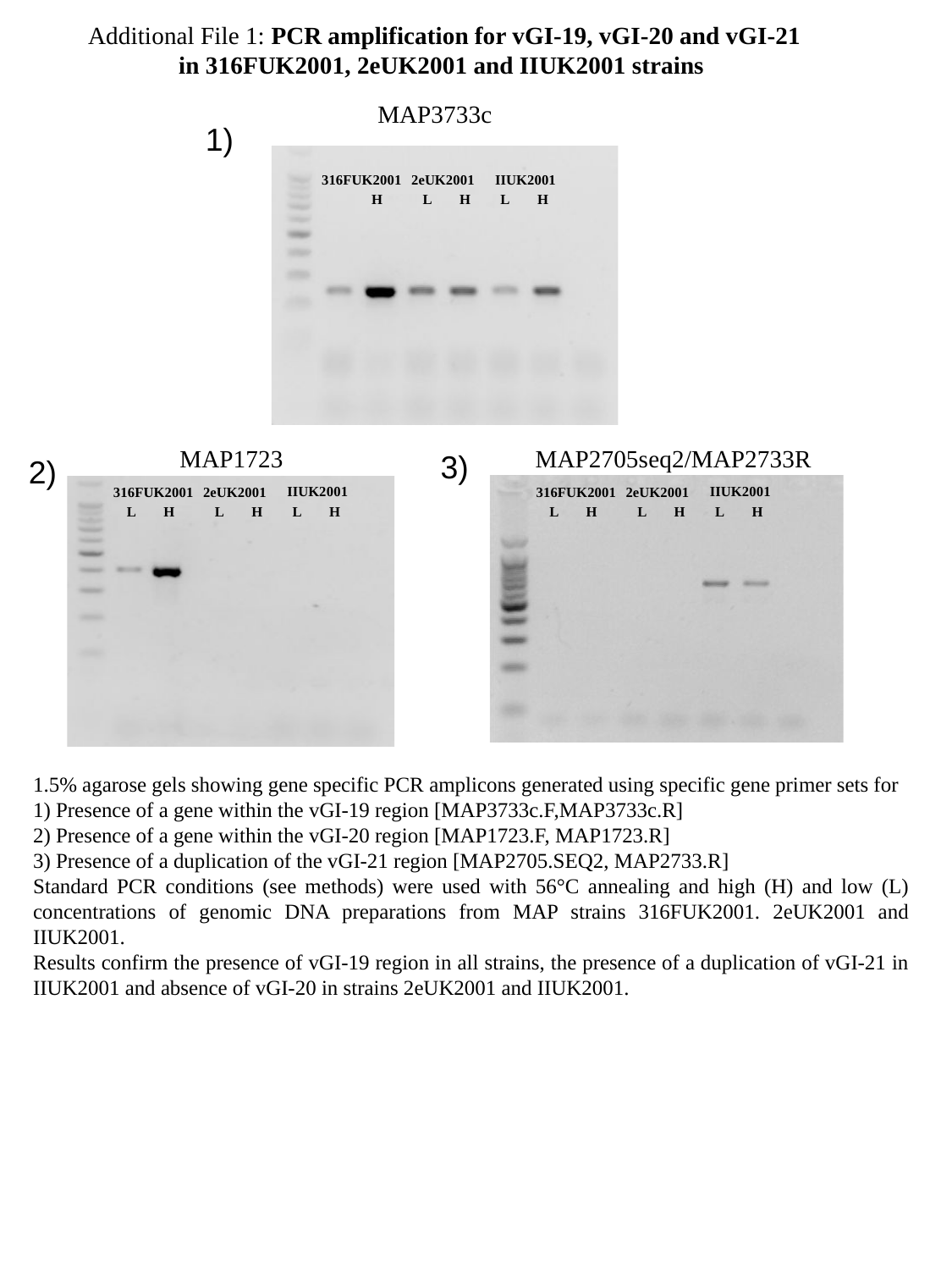

Additional File 1: PCR amplification for vGI-19, vGI-20 and vGI-21 in 316FUK2001, 2eUK2001 and IIUK2001 strains
MAP3733c
1)
IIUK2001
316FUK2001
2eUK2001
H
L
H
L
H
MAP2705seq2/MAP2733R
MAP1723
3)
2)
IIUK2001
IIUK2001
316FUK2001
2eUK2001
316FUK2001
2eUK2001
L
H
L
H
L
H
L
H
L
H
L
H
1.5% agarose gels showing gene specific PCR amplicons generated using specific gene primer sets for
1) Presence of a gene within the vGI-19 region [MAP3733c.F,MAP3733c.R]
2) Presence of a gene within the vGI-20 region [MAP1723.F, MAP1723.R]
3) Presence of a duplication of the vGI-21 region [MAP2705.SEQ2, MAP2733.R]
Standard PCR conditions (see methods) were used with 56°C annealing and high (H) and low (L) concentrations of genomic DNA preparations from MAP strains 316FUK2001. 2eUK2001 and IIUK2001.
Results confirm the presence of vGI-19 region in all strains, the presence of a duplication of vGI-21 in IIUK2001 and absence of vGI-20 in strains 2eUK2001 and IIUK2001.
