# Supplementary material for: Genomic variations associated with attenuation in Mycobacterium avium subsp. paratuberculosis vaccine strains
Source: BMC Microbiol. 2013 Jan 22;13:11. doi: 10.1186/1471-2180-13-11 (PMC3599157; doi:10.1186/1471-2180-13-11)
Supplement: Additional file 2 — Mouse Model Data File. Tables and statistical analyses of virulence experiments in mice. (DOCX 86 kb) [file 1471-2180-13-11-S2.docx]

**Additional File 1**

1. **Analysis of Body Weight**

Fitting a Linear Mixed Model to these data gives the following output:

REML variance components analysis

Response variate: BODY_WT

Fixed model: Constant + Strain + Week + Strain.Week

Number of units: 148

Residual term has been added to model

Sparse algorithm with AI optimisation

Residual variance model

Term Factor Model(order) Parameter Estimate s.e.

Residual Identity Sigma2 4.593 0.563

Tests for fixed effects

Sequentially adding terms to fixed model

Fixed term Wald statistic n.d.f. F statistic d.d.f. F pr

Strain 3.60 4 0.90 133.0 0.466

Week 120.81 2 60.41 133.0 <0.001

Strain.Week 13.54 8 1.69 133.0 0.106

Dropping individual terms from full fixed model

Fixed term Wald statistic n.d.f. F statistic d.d.f. F pr

Strain.Week 13.54 8 1.69 133.0 0.106

*Message: denominator degrees of freedom for approximate F-tests are calculated using algebraic derivatives ignoring fixed/boundary/singular variance parameters.*

Table of effects for Constant

26.87 Standard error: 0.678

Table of effects for Strain

Strain 107 2e 316F II PBS

0.0000 -0.8304 0.7307 0.9692 0.6349

Standard errors

Strain 107 2e 316F II PBS

0.0000 0.9585 0.9585 0.9585 0.9585

Table of effects for Week

Week 4 8 12

0.000 3.371 5.342

Standard errors

Week 4 8 12

0.000 0.958 0.985

Standard errors

Average: 0.9716

Maximum: 0.9848

Minimum: 0.9585

Table of effects for Strain.Week

Week 4 8 12

Strain

107 0.0000 0.0000 0.0000

2e 0.0000 -0.1360 1.2837

316F 0.0000 -1.5837 -2.8005

II 0.0000 -1.3602 -1.3957

PBS 0.0000 -1.7803 0.1140

Standard errors

Week 4 8 12

Strain

107 0.0000 0.0000 0.0000

2e 0.0000 1.3555 1.3926

316F 0.0000 1.3555 1.3742

II 0.0000 1.3555 1.3742

PBS 0.0000 1.3555 1.3742

Standard errors

Average: 1.367

Maximum: 1.393

Minimum: 1.356

Table of predicted means for Constant

29.56 Standard error: 0.176

Table of predicted means for Strain

Strain 107 2e 316F II PBS

29.77 29.32 29.04 29.82 29.85

Standard errors

Strain 107 2e 316F II PBS

0.40 0.40 0.39 0.39 0.39

Standard errors

Average: 0.3942

Maximum: 0.3985

Minimum: 0.3913

Table of predicted means for Week

Week 4 8 12

27.17 29.56 31.95

Standard errors

Week 4 8 12

0.30 0.30 0.31

Standard errors

Average: 0.3053

Maximum: 0.3098

Minimum: 0.3031

Table of predicted means for Strain.Week

Week 4 8 12

Strain

107 26.87 30.24 32.21

2e 26.03 29.27 32.66

316F 27.60 29.38 30.14

II 27.83 29.85 31.78

PBS 27.50 29.09 32.96

Standard errors

Week 4 8 12

Strain

107 0.68 0.68 0.71

2e 0.68 0.68 0.71

316F 0.68 0.68 0.68

II 0.68 0.68 0.68

PBS 0.68 0.68 0.68

Standard errors

Average: 0.6826

Maximum: 0.7144

Minimum: 0.6778

The formal tests show differences in mean body weight associated with week (p<0.001), but none associated with strain (p=0.466) or with the week by Strain interaction (p=0.106), which would represent differences in time trend for different strains.

1. **Analysis of Rank of Spleen Weight as a Percentage of Body Weight**

To aid in interpretation of the models of ranked data, we tabulate the means for the unranked data:

Week 4 8

Nobservd Mean Nobservd Mean

Strain

107 10 0.5657 10 0.5185

2e 10 0.3648 10 0.3526

316F 10 0.3506 10 0.3402

II 10 0.3417 10 0.3827

PBS 10 0.3387 10 0.2825

Week 12

Nobservd Mean

Strain

107 9 0.3738

2e 9 0.3508

316F 10 0.4097

II 10 0.3585

PBS 10 0.3014

Because of an unusual distribution of observed values in these data, they have been analysed using the ranks of the data in a Linear Mixed Model. The output is as follows:

REML variance components analysis

Response variate: Spleen_Rank

Fixed model: Constant + Strain + Week + Strain.Week

Number of units: 148

Residual term has been added to model

Sparse algorithm with AI optimisation

Residual variance model

Term Factor Model(order) Parameter Estimate s.e.

Residual Identity Sigma2 1410. 173.

Tests for fixed effects

Sequentially adding terms to fixed model

Fixed term Wald statistic n.d.f. F statistic d.d.f. F pr

Strain 35.34 4 8.83 133.0 <0.001

Week 0.43 2 0.21 133.0 0.807

Strain.Week 22.80 8 2.85 133.0 0.006

Dropping individual terms from full fixed model

Fixed term Wald statistic n.d.f. F statistic d.d.f. F pr

Strain.Week 22.80 8 2.85 133.0 0.006

*Message: denominator degrees of freedom for approximate F-tests are calculated using algebraic derivatives ignoring fixed/boundary/singular variance parameters.*

Table of effects for Constant

75.10 Standard error: 11.875

Table of effects for Strain

Strain 107 2e 316F II PBS

0.000 13.300 -0.700 -9.500 -10.000

Standard errors

Strain 107 2e 316F II PBS

0.000 16.794 16.794 16.794 16.794

Table of effects for Week

Week 4 8 12

0.00 37.60 20.12

Standard errors

Week 4 8 12

0.00 16.79 17.25

Standard errors

Average: 17.02

Maximum: 17.25

Minimum: 16.79

Table of effects for Strain.Week

Week 4 8 12

Strain

107 0.00 0.00 0.00

2e 0.00 -49.20 -40.08

316F 0.00 -44.50 14.48

II 0.00 -14.20 -9.42

PBS 0.00 -84.50 -48.02

Standard errors

Week 4 8 12

Strain

107 0.00 0.00 0.00

2e 0.00 23.75 24.40

316F 0.00 23.75 24.08

II 0.00 23.75 24.08

PBS 0.00 23.75 24.08

Standard errors

Average: 23.95

Maximum: 24.40

Minimum: 23.75

Table of predicted means for Constant

74.60 Standard error: 3.089

Table of predicted means for Strain

Strain 107 2e 316F II PBS

94.34 77.88 83.63 76.97 40.17

Standard errors

Strain 107 2e 316F II PBS

6.98 6.98 6.86 6.86 6.86

Standard errors

Average: 6.906

Maximum: 6.982

Minimum: 6.856

Table of predicted means for Week

Week 4 8 12

73.72 72.84 77.23

Standard errors

Week 4 8 12

5.31 5.31 5.43

Standard errors

Average: 5.350

Maximum: 5.427

Minimum: 5.311

Table of predicted means for Strain.Week

Week 4 8 12

Strain

107 75.10 112.70 95.22

2e 88.40 76.80 68.44

316F 74.40 67.50 109.00

II 65.60 89.00 76.30

PBS 65.10 18.20 37.20

Standard errors

Week 4 8 12

Strain

107 11.87 11.87 12.52

2e 11.87 11.87 12.52

316F 11.87 11.87 11.87

II 11.87 11.87 11.87

PBS 11.87 11.87 11.87

Standard errors

Average: 11.96

Maximum: 12.52

Minimum: 11.87

There is highly statistically significant evidence of differences in the pattern of means in successive weeks for different strains (p=0.006). The means for MAP infected animals are consistently higher than those for PBS animals.

Focusing only on responses from MAP infected animals, the model output is as follows:

REML variance components analysis

Response variate: Spleen_Rank

Fixed model: Constant + Strain + Week + Strain.Week

Number of units: 118

Residual term has been added to model

Sparse algorithm with AI optimisation

Analysis is subject to the restriction on Spleen_Rank

Residual variance model

Term Factor Model(order) Parameter Estimate s.e.

Residual Identity Sigma2 1094. 150.

Tests for fixed effects

Sequentially adding terms to fixed model

Fixed term Wald statistic n.d.f. F statistic d.d.f. F pr

Strain 4.56 3 1.52 106.0 0.214

Week 1.83 2 0.91 106.0 0.404

Strain.Week 12.73 6 2.12 106.0 0.057

Dropping individual terms from full fixed model

Fixed term Wald statistic n.d.f. F statistic d.d.f. F pr

Strain.Week 12.73 6 2.12 106.0 0.057

*Message: denominator degrees of freedom for approximate F-tests are calculated using algebraic derivatives ignoring fixed/boundary/singular variance parameters.*

Table of effects for Constant

54.70 Standard error: 10.461

Table of effects for Strain

Strain 107 2e 316F II PBS

0.000 8.900 -1.200 -11.100 *

Standard errors

Strain 107 2e 316F II PBS

0.000 14.794 14.794 14.794 *

Table of effects for Week

Week 4 8 12

0.00 30.80 15.08

Standard errors

Week 4 8 12

0.00 14.79 15.20

Standard errors

Average: 15.00

Maximum: 15.20

Minimum: 14.79

Table of effects for Strain.Week

Week 4 8 12

Strain

107 0.00 0.00 0.00

2e 0.00 -41.30 -32.34

316F 0.00 -39.60 13.52

II 0.00 -10.40 -5.88

PBS * * *

Standard errors

Week 4 8 12

Strain

107 0.00 0.00 0.00

2e 0.00 20.92 21.49

316F 0.00 20.92 21.21

II 0.00 20.92 21.21

PBS * * *

Standard errors

Average: 21.11

Maximum: 21.49

Minimum: 20.92

Table of predicted means for Constant

59.48 Standard error: 3.048

Table of predicted means for Strain

Strain 107 2e 316F II PBS

69.99 54.34 60.10 53.47 *

Standard errors

Strain 107 2e 316F II PBS

6.15 6.15 6.04 6.04 *

Standard errors

Average: 6.095

Maximum: 6.150

Minimum: 6.040

Table of predicted means for Week

Week 4 8 12

* * *

All values in table missing: due to missing factor combinations in higher order interactions.

Table of predicted means for Strain.Week

Week 4 8 12

Strain

107 54.70 85.50 69.78

2e 63.60 53.10 46.33

316F 53.50 44.70 82.10

II 43.60 64.00 52.80

PBS * * *

Standard errors

Week 4 8 12

Strain

107 10.46 10.46 11.03

2e 10.46 10.46 11.03

316F 10.46 10.46 10.46

II 10.46 10.46 10.46

PBS * * *

Standard errors

Average: 10.56

Maximum: 11.03

Minimum: 10.46

There is no formal statistical evidence of any differences between the different MAP strains (p=0.057).

1. **Analysis of Liver Weight as a Percentage of Body Weight**

Fitting a Linear Mixed Model to these data gives the following output:

REML variance components analysis

Response variate: LIV_WT_%_BWT

Fixed model: Constant + Strain + Week + Strain.Week

Number of units: 148

Residual term has been added to model

Sparse algorithm with AI optimisation

Residual variance model

Term Factor Model(order) Parameter Estimate s.e.

Residual Identity Sigma2 0.394 0.0484

Tests for fixed effects

Sequentially adding terms to fixed model

Fixed term Wald statistic n.d.f. F statistic d.d.f. F pr

Strain 12.35 4 3.09 133.0 0.018

Week 2.51 2 1.25 133.0 0.289

Strain.Week 9.28 8 1.16 133.0 0.328

Dropping individual terms from full fixed model

Fixed term Wald statistic n.d.f. F statistic d.d.f. F pr

Strain.Week 9.28 8 1.16 133.0 0.328

*Message: denominator degrees of freedom for approximate F-tests are calculated using algebraic derivatives ignoring fixed/boundary/singular variance parameters.*

Table of effects for Constant

5.216 Standard error: 0.1986

Table of effects for Strain

Strain 107 2e 316F II PBS

0.0000 0.0794 -0.1873 0.1741 -0.5637

Standard errors

Strain 107 2e 316F II PBS

0.0000 0.2808 0.2808 0.2808 0.2808

Table of effects for Week

Week 4 8 12

0.0000 0.2414 -0.0994

Standard errors

Week 4 8 12

0.0000 0.2808 0.2885

Standard errors

Average: 0.2847

Maximum: 0.2885

Minimum: 0.2808

Table of effects for Strain.Week

Week 4 8 12

Strain

107 0.0000 0.0000 0.0000

2e 0.0000 -0.3889 -0.3438

316F 0.0000 -0.3268 0.0378

II 0.0000 -0.9101 -0.5089

PBS 0.0000 -0.2591 0.3113

Standard errors

Week 4 8 12

Strain

107 0.0000 0.0000 0.0000

2e 0.0000 0.3972 0.4080

316F 0.0000 0.3972 0.4026

II 0.0000 0.3972 0.4026

PBS 0.0000 0.3972 0.4026

Standard errors

Average: 0.4006

Maximum: 0.4080

Minimum: 0.3972

Table of predicted means for Constant

5.004 Standard error: 0.0517

Table of predicted means for Strain

Strain 107 2e 316F II PBS

5.263 5.098 4.979 4.964 4.717

Standard errors

Strain 107 2e 316F II PBS

0.117 0.117 0.115 0.115 0.115

Standard errors

Average: 0.1155

Maximum: 0.1168

Minimum: 0.1146

Table of predicted means for Week

Week 4 8 12

5.116 4.981 4.916

Standard errors

Week 4 8 12

0.089 0.089 0.091

Standard errors

Average: 0.08946

Maximum: 0.09076

Minimum: 0.08881

Table of predicted means for Strain.Week

Week 4 8 12

Strain

107 5.216 5.457 5.116

2e 5.295 5.148 4.852

316F 5.028 4.943 4.967

II 5.390 4.721 4.781

PBS 4.652 4.634 4.864

Standard errors

Week 4 8 12

Strain

107 0.199 0.199 0.209

2e 0.199 0.199 0.209

316F 0.199 0.199 0.199

II 0.199 0.199 0.199

PBS 0.199 0.199 0.199

Standard errors

Average: 0.2000

Maximum: 0.2093

Minimum: 0.1986

There is clear statistical evidence of a difference in means associated with Strain (p=0.018). Examination of the means suggests that the major factor in this is the difference between PBS animals and those infected with MAP.

Fitting the model without PBS animals gives the following results:

REML variance components analysis

Response variate: LIV_WT_%_BWT

Fixed model: Constant + Strain + Week + Strain.Week

Number of units: 118

Residual term has been added to model

Sparse algorithm with AI optimisation

Analysis is subject to the restriction on LIV_WT_%_BWT

Residual variance model

Term Factor Model(order) Parameter Estimate s.e.

Residual Identity Sigma2 0.408 0.0560

Tests for fixed effects

Sequentially adding terms to fixed model

Fixed term Wald statistic n.d.f. F statistic d.d.f. F pr

Strain 4.30 3 1.43 106.0 0.237

Week 4.34 2 2.17 106.0 0.119

Strain.Week 6.27 6 1.04 106.0 0.401

Dropping individual terms from full fixed model

Fixed term Wald statistic n.d.f. F statistic d.d.f. F pr

Strain.Week 6.27 6 1.04 106.0 0.401

*Message: denominator degrees of freedom for approximate F-tests are calculated using algebraic derivatives ignoring fixed/boundary/singular variance parameters.*

Table of effects for Constant

5.216 Standard error: 0.2019

Table of effects for Strain

Strain 107 2e 316F II PBS

0.0000 0.0794 -0.1873 0.1741 *

Standard errors

Strain 107 2e 316F II PBS

0.0000 0.2855 0.2855 0.2855 *

Table of effects for Week

Week 4 8 12

0.0000 0.2414 -0.0994

Standard errors

Week 4 8 12

0.0000 0.2855 0.2933

Standard errors

Average: 0.2894

Maximum: 0.2933

Minimum: 0.2855

Table of effects for Strain.Week

Week 4 8 12

Strain

107 0.0000 0.0000 0.0000

2e 0.0000 -0.3889 -0.3438

316F 0.0000 -0.3268 0.0378

II 0.0000 -0.9101 -0.5089

PBS * * *

Standard errors

Week 4 8 12

Strain

107 0.0000 0.0000 0.0000

2e 0.0000 0.4037 0.4148

316F 0.0000 0.4037 0.4093

II 0.0000 0.4037 0.4093

PBS * * *

Standard errors

Average: 0.4074

Maximum: 0.4148

Minimum: 0.4037

Table of predicted means for Constant

5.076 Standard error: 0.0588

Table of predicted means for Strain

Strain 107 2e 316F II PBS

5.263 5.098 4.979 4.964 *

Standard errors

Strain 107 2e 316F II PBS

0.119 0.119 0.117 0.117 *

Standard errors

Average: 0.1176

Maximum: 0.1187

Minimum: 0.1165

Table of predicted means for Week

Week 4 8 12

* * *

All values in table missing: due to missing factor combinations in higher order interactions.

Table of predicted means for Strain.Week

Week 4 8 12

Strain

107 5.216 5.457 5.116

2e 5.295 5.148 4.852

316F 5.028 4.943 4.967

II 5.390 4.721 4.781

PBS * * *

Standard errors

Week 4 8 12

Strain

107 0.202 0.202 0.213

2e 0.202 0.202 0.213

316F 0.202 0.202 0.202

II 0.202 0.202 0.202

PBS * * *

Standard errors

Average: 0.2037

Maximum: 0.2128

Minimum: 0.2019

There is no statistical evidence for any difference in the mean responses for different strains in different weeks. To summarise these latter results we will refit the model purely in terms of the strain by week interaction:

REML variance components analysis

Response variate: LIV_WT_%_BWT

Fixed model: Constant + Strain.Week

Number of units: 118

Residual term has been added to model

Sparse algorithm with AI optimisation

Analysis is subject to the restriction on LIV_WT_%_BWT

Residual variance model

Term Factor Model(order) Parameter Estimate s.e.

Residual Identity Sigma2 0.408 0.0560

Tests for fixed effects

Sequentially adding terms to fixed model

Fixed term Wald statistic n.d.f. F statistic d.d.f. F pr

Strain.Week 14.91 11 1.36 106.0 0.205

Dropping individual terms from full fixed model

Fixed term Wald statistic n.d.f. F statistic d.d.f. F pr

Strain.Week 14.91 11 1.36 106.0 0.205

*Message: denominator degrees of freedom for approximate F-tests are calculated using algebraic derivatives ignoring fixed/boundary/singular variance parameters.*

Table of effects for Constant

5.216 Standard error: 0.2019

Table of effects for Strain.Week

Week 4 8 12

Strain

107 0.0000 0.2414 -0.0994

2e 0.0794 -0.0681 -0.3639

316F -0.1873 -0.2727 -0.2490

II 0.1741 -0.4946 -0.4342

PBS * * *

Standard errors

Week 4 8 12

Strain

107 0.0000 0.2855 0.2933

2e 0.2855 0.2855 0.2933

316F 0.2855 0.2855 0.2855

II 0.2855 0.2855 0.2855

PBS * * *

Standard errors

Average: 0.2869

Maximum: 0.2933

Minimum: 0.2855

Table of predicted means for Constant

5.076 Standard error: 0.0588

Table of predicted means for Strain.Week

Week 4 8 12

Strain

107 5.216 5.457 5.116

2e 5.295 5.148 4.852

316F 5.028 4.943 4.967

II 5.390 4.721 4.781

PBS * * *

Standard errors

Week 4 8 12

Strain

107 0.202 0.202 0.213

2e 0.202 0.202 0.213

316F 0.202 0.202 0.202

II 0.202 0.202 0.202

PBS * * *

Standard errors

Average: 0.2037

Maximum: 0.2128

Minimum: 0.2019

With p=0.2, there is no evidence of any strain or week effects.

1. **Analysis of Mean Bacterial Counts**

Fitting a Linear Model to the mean bacterial count data is inappropriate because of the large number of zeros in the dataset. A Linear Model is therefore fitted to the ranks of the means, giving the following output:

REML variance components analysis

Response variate: Count_Rank

Fixed model: Constant + Strain_Count + Week_Count + Strain_Count.Week_Count

Number of units: 148

Residual term has been added to model

Sparse algorithm with AI optimisation

Residual variance model

Term Factor Model(order) Parameter Estimate s.e.

Residual Identity Sigma2 255.3 31.3

Tests for fixed effects

Sequentially adding terms to fixed model

Fixed term Wald statistic n.d.f. F statistic d.d.f. F pr

Strain_Count 667.83 4 166.96 133.0 <0.001

Week_Count 64.88 2 32.44 133.0 <0.001

Strain_Count.Week_Count 93.90 8 11.74 133.0 <0.001

Dropping individual terms from full fixed model

Fixed term Wald statistic n.d.f. F statistic d.d.f. F pr

Strain_Count.Week_Count 93.90 8 11.74 133.0 <0.001

*Message: denominator degrees of freedom for approximate F-tests are calculated using algebraic derivatives ignoring fixed/boundary/singular variance parameters.*

Table of effects for Constant

123.1 Standard error: 5.05

Table of effects for Strain_Count

Strain_Count 107 2e 316F II PBS

0.00 -48.30 -36.15 -9.00 -89.15

Standard errors

Strain_Count 107 2e 316F II PBS

0.00 7.15 7.15 7.15 7.15

Table of effects for Week_Count

Week_Count 4 8 12

0.000 12.850 16.739

Standard errors

Week_Count 4 8 12

0.000 7.146 7.342

Standard errors

Average: 7.244

Maximum: 7.342

Minimum: 7.146

Table of effects for Strain_Count.Week_Count

Week_Count 4 8 12

Strain_Count

107 0.00 0.00 0.00

2e 0.00 -46.40 -53.53

316F 0.00 -21.65 -69.74

II 0.00 -35.20 -70.89

PBS 0.00 -12.85 -16.74

Standard errors

Week_Count 4 8 12

Strain_Count

107 0.00 0.00 0.00

2e 0.00 10.11 10.38

316F 0.00 10.11 10.25

II 0.00 10.11 10.25

PBS 0.00 10.11 10.25

Standard errors

Average: 10.19

Maximum: 10.38

Minimum: 10.11

Table of predicted means for Constant

74.69 Standard error: 1.314

Table of predicted means for Strain_Count

Strain_Count 107 2e 316F II PBS

133.01 51.40 66.40 88.65 34.00

Standard errors

Strain_Count 107 2e 316F II PBS

2.97 2.97 2.92 2.92 2.92

Standard errors

Average: 2.939

Maximum: 2.971

Minimum: 2.917

Table of predicted means for Week_Count

Week_Count 4 8 12

86.63 76.26 61.19

Standard errors

Week_Count 4 8 12

2.26 2.26 2.31

Standard errors

Average: 2.276

Maximum: 2.310

Minimum: 2.260

Table of predicted means for Strain_Count.Week_Count

Week_Count 4 8 12

Strain_Count

107 123.15 136.00 139.89

2e 74.85 41.30 38.06

316F 87.00 78.20 34.00

II 114.15 91.80 60.00

PBS 34.00 34.00 34.00

Standard errors

Week_Count 4 8 12

Strain_Count

107 5.05 5.05 5.33

2e 5.05 5.05 5.33

316F 5.05 5.05 5.05

II 5.05 5.05 5.05

PBS 5.05 5.05 5.05

Standard errors

Average: 5.090

Maximum: 5.327

Minimum: 5.053

There is highly statistically significant evidence of differences in the pattern of mean ranks over time for different strains (p<0.001). The PBS group is particularly anomalous, since no bacteria are ever sampled from it since it is a negative control. Excluding samples from the PBS group and refitting the model gives:

REML variance components analysis

Response variate: Count_Rank

Fixed model: Constant + Strain_Count + Week_Count + Strain_Count.Week_Count

Number of units: 118

Residual term has been added to model

Sparse algorithm with AI optimisation

Analysis is subject to the restriction on Count_Rank

Residual variance model

Term Factor Model(order) Parameter Estimate s.e.

Residual Identity Sigma2 199.8 27.4

Tests for fixed effects

Sequentially adding terms to fixed model

Fixed term Wald statistic n.d.f. F statistic d.d.f. F pr

Strain_Count 421.60 3 140.53 106.0 <0.001

Week_Count 64.65 2 32.32 106.0 <0.001

Strain_Count.Week_Count 71.79 6 11.96 106.0 <0.001

Dropping individual terms from full fixed model

Fixed term Wald statistic n.d.f. F statistic d.d.f. F pr

Strain_Count.Week_Count 71.79 6 11.96 106.0 <0.001

*Message: denominator degrees of freedom for approximate F-tests are calculated using algebraic derivatives ignoring fixed/boundary/singular variance parameters.*

Table of effects for Constant

93.15 Standard error: 4.469

Table of effects for Strain_Count

Strain_Count 107 2e 316F II PBS

0.00 -42.30 -34.65 -9.00 *

Standard errors

Strain_Count 107 2e 316F II PBS

0.00 6.32 6.32 6.32 *

Table of effects for Week_Count

Week_Count 4 8 12

0.000 12.850 16.739

Standard errors

Week_Count 4 8 12

0.000 6.321 6.494

Standard errors

Average: 6.407

Maximum: 6.494

Minimum: 6.321

Table of effects for Strain_Count.Week_Count

Week_Count 4 8 12

Strain_Count

107 0.00 0.00 0.00

2e 0.00 -40.40 -46.20

316F 0.00 -20.15 -56.24

II 0.00 -35.20 -64.89

PBS * * *

Standard errors

Week_Count 4 8 12

Strain_Count

107 0.00 0.00 0.00

2e 0.00 8.94 9.18

316F 0.00 8.94 9.06

II 0.00 8.94 9.06

PBS * * *

Standard errors

Average: 9.021

Maximum: 9.184

Minimum: 8.939

Table of predicted means for Constant

59.60 Standard error: 1.302

Table of predicted means for Strain_Count

Strain_Count 107 2e 316F II PBS

103.01 31.85 42.90 60.65 *

Standard errors

Strain_Count 107 2e 316F II PBS

2.63 2.63 2.58 2.58 *

Standard errors

Average: 2.604

Maximum: 2.628

Minimum: 2.580

Table of predicted means for Week_Count

Week_Count 4 8 12

* * *

All values in table missing: due to missing factor combinations in higher order interactions.

Table of predicted means for Strain_Count.Week_Count

Week_Count 4 8 12

Strain_Count

107 93.15 106.00 109.89

2e 50.85 23.30 21.39

316F 58.50 51.20 19.00

II 84.15 61.80 36.00

PBS * * *

Standard errors

Week_Count 4 8 12

Strain_Count

107 4.47 4.47 4.71

2e 4.47 4.47 4.71

316F 4.47 4.47 4.47

II 4.47 4.47 4.47

PBS * * *

Standard errors

Average: 4.510

Maximum: 4.711

Minimum: 4.469

Again we see highly statistically significant differences in the mean ranks over time for different groups (p<0.001). Excluding the wildtype samples, and hence just comparing the vaccine strains gives:

REML variance components analysis

Response variate: Count_Rank

Fixed model: Constant + Strain_Count + Week_Count + Strain_Count.Week_Count

Number of units: 89

Residual term has been added to model

Sparse algorithm with AI optimisation

Analysis is subject to the restriction on Count_Rank

Residual variance model

Term Factor Model(order) Parameter Estimate s.e.

Residual Identity Sigma2 222.3 35.2

Tests for fixed effects

Sequentially adding terms to fixed model

Fixed term Wald statistic n.d.f. F statistic d.d.f. F pr

Strain_Count 53.26 2 26.63 80.0 <0.001

Week_Count 99.07 2 49.53 80.0 <0.001

Strain_Count.Week_Count 12.71 4 3.18 80.0 0.018

Dropping individual terms from full fixed model

Fixed term Wald statistic n.d.f. F statistic d.d.f. F pr

Strain_Count.Week_Count 12.71 4 3.18 80.0 0.018

*Message: denominator degrees of freedom for approximate F-tests are calculated using algebraic derivatives ignoring fixed/boundary/singular variance parameters.*

Table of effects for Constant

50.35 Standard error: 4.715

Table of effects for Strain_Count

Strain_Count 107 2e 316F II PBS

* 0.00 7.45 32.80 *

Standard errors

Strain_Count 107 2e 316F II PBS

* 0.00 6.67 6.67 *

Table of effects for Week_Count

Week_Count 4 8 12

0.00 -27.05 -28.96

Standard errors

Week_Count 4 8 12

0.00 6.67 6.85

Standard errors

Average: 6.760

Maximum: 6.851

Minimum: 6.668

Table of effects for Strain_Count.Week_Count

Week_Count 4 8 12

Strain_Count

107 * * *

2e 0.000 0.000 0.000

316F 0.000 19.950 -9.839

II 0.000 4.950 -18.289

PBS * * *

Standard errors

Week_Count 4 8 12

Strain_Count

107 * * *

2e 0.000 0.000 0.000

316F 0.000 9.430 9.561

II 0.000 9.430 9.561

PBS * * *

Standard errors

Average: 9.496

Maximum: 9.561

Minimum: 9.430

Table of predicted means for Constant

44.74 Standard error: 1.581

Table of predicted means for Strain_Count

Strain_Count 107 2e 316F II PBS

* 31.68 42.50 60.03 *

Standard errors

Strain_Count 107 2e 316F II PBS

* 2.77 2.72 2.72 *

Standard errors

Average: 2.739

Maximum: 2.772

Minimum: 2.722

Table of predicted means for Week_Count

Week_Count 4 8 12

* * *

All values in table missing: due to missing factor combinations in higher order interactions.

Table of predicted means for Strain_Count.Week_Count

Week_Count 4 8 12

Strain_Count

107 * * *

2e 50.35 23.30 21.39

316F 57.80 50.70 19.00

II 83.15 61.05 35.90

PBS * * *

Standard errors

Week_Count 4 8 12

Strain_Count

107 * * *

2e 4.72 4.72 4.97

316F 4.72 4.72 4.72

II 4.72 4.72 4.72

PBS * * *

Standard errors

Average: 4.744

Maximum: 4.970

Minimum: 4.715

We detect statistically significant (p=0.018) differences in the mean ranks over time for different vaccine strains.

1. **Analysis of mononuclear leucocyte clusters (LCA)**

To aid in interpretation of the models of ranked data, we tabulate the means for the unranked data:

Week 4 8

Nobservd Mean Nobservd Mean

Strain

107 10 1.5247 10 2.0351

2e 10 1.1970 10 1.2822

316F 10 0.9054 10 0.6739

II 10 0.8215 10 0.9512

PBS 10 0.0740 10 0.0513

Week 12

Nobservd Mean

Strain

107 9 1.6781

2e 9 0.8777

316F 10 0.7078

II 10 0.9249

PBS 10 0.0386

Because of an unusual distribution of observed values in these data, they have been analysed using the ranks of the data in a Linear Mixed Model. Fitting the model to the MAP groups alone, the output is as follows:

REML variance components analysis

Response variate: LCA_Rank2

Fixed model: Constant + Week + Strain + Week.Strain

Number of units: 118

Residual term has been added to model

Sparse algorithm with AI optimisation

Analysis is subject to the restriction on LCA_Rank2

Residual variance model

Term Factor Model(order) Parameter Estimate s.e.

Residual Identity Sigma2 954.1 131.1

Tests for fixed effects

Sequentially adding terms to fixed model

Fixed term Wald statistic n.d.f. F statistic d.d.f. F pr

Week 1.62 2 0.81 106.0 0.448

Strain 27.34 3 9.11 106.0 <0.001

Week.Strain 8.53 6 1.42 106.0 0.213

Dropping individual terms from full fixed model

Fixed term Wald statistic n.d.f. F statistic d.d.f. F pr

Week.Strain 8.53 6 1.42 106.0 0.213

*Message: denominator degrees of freedom for approximate F-tests are calculated using algebraic derivatives ignoring fixed/boundary/singular variance parameters.*

Table of effects for Constant

61.70 Standard error: 9.768

Table of effects for Week

Week 4 8 12

0.00 33.60 28.69

Standard errors

Week 4 8 12

0.00 13.81 14.19

Standard errors

Average: 14.00

Maximum: 14.19

Minimum: 13.81

Table of effects for Strain

Strain 107 2e 316F II PBS

0.000 4.850 -13.400 -14.950 *

Standard errors

Strain 107 2e 316F II PBS

0.000 13.814 13.814 13.814 *

Table of effects for Week.Strain

Strain 107 2e 316F II PBS

Week

4 0.00 0.00 0.00 0.00 *

8 0.00 -30.60 -44.35 -25.30 *

12 0.00 -44.91 -37.39 -20.34 *

Standard errors

Strain 107 2e 316F II PBS

Week

4 0.00 0.00 0.00 0.00 *

8 0.00 19.54 19.54 19.54 *

12 0.00 20.07 19.80 19.80 *

Standard errors

Average: 19.71

Maximum: 20.07

Minimum: 19.54

Table of predicted means for Constant

59.68 Standard error: 2.846

Table of predicted means for Week

Week 4 8 12

* * *

All values in table missing: due to missing factor combinations in higher order interactions.

Table of predicted means for Strain

Strain 107 2e 316F II PBS

82.46 62.14 41.82 52.30 *

Standard errors

Strain 107 2e 316F II PBS

5.74 5.74 5.64 5.64 *

Standard errors

Average: 5.691

Maximum: 5.743

Minimum: 5.639

Table of predicted means for Week.Strain

Strain 107 2e 316F II PBS

Week

4 61.70 66.55 48.30 46.75 *

8 95.30 69.55 37.55 55.05 *

12 90.39 50.33 39.60 55.10 *

Standard errors

Strain 107 2e 316F II PBS

Week

4 9.77 9.77 9.77 9.77 *

8 9.77 9.77 9.77 9.77 *

12 10.30 10.30 9.77 9.77 *

Standard errors

Average: 9.856

Maximum: 10.30

Minimum: 9.768

There is a highly statistically significant (p<0.001) difference in the mean rank densities for different strains. There is no evidence that these means differ for different weeks or that different strains behave differently in different weeks. The biggest difference is between the mean for the wildtype strain and all the others. Refitting the model to only the vaccine strains gives:

REML variance components analysis

Response variate: LCA_Rank2

Fixed model: Constant + Week + Strain + Week.Strain

Number of units: 89

Residual term has been added to model

Sparse algorithm with AI optimisation

Analysis is subject to the restriction on LCA_Rank2

Residual variance model

Term Factor Model(order) Parameter Estimate s.e.

Residual Identity Sigma2 647.1 102.3

Tests for fixed effects

Sequentially adding terms to fixed model

Fixed term Wald statistic n.d.f. F statistic d.d.f. F pr

Week 0.57 2 0.29 80.0 0.752

Strain 7.48 2 3.74 80.0 0.028

Week.Strain 2.71 4 0.68 80.0 0.610

Dropping individual terms from full fixed model

Fixed term Wald statistic n.d.f. F statistic d.d.f. F pr

Week.Strain 2.71 4 0.68 80.0 0.610

*Message: denominator degrees of freedom for approximate F-tests are calculated using algebraic derivatives ignoring fixed/boundary/singular variance parameters.*

Table of effects for Constant

57.70 Standard error: 8.044

Table of effects for Week

Week 4 8 12

0.000 1.550 -13.811

Standard errors

Week 4 8 12

0.000 11.376 11.688

Standard errors

Average: 11.53

Maximum: 11.69

Minimum: 11.38

Table of effects for Strain

Strain 107 2e 316F II PBS

* 0.00 -17.00 -17.00 *

Standard errors

Strain 107 2e 316F II PBS

* 0.00 11.38 11.38 *

Table of effects for Week.Strain

Strain 107 2e 316F II PBS

Week

4 * 0.000 0.000 0.000 *

8 * 0.000 -9.750 5.400 *

12 * 0.000 7.211 21.511 *

Standard errors

Strain 107 2e 316F II PBS

Week

4 * 0.000 0.000 0.000 *

8 * 0.000 16.089 16.089 *

12 * 0.000 16.311 16.311 *

Standard errors

Average: 16.20

Maximum: 16.31

Minimum: 16.09

Table of predicted means for Constant

44.99 Standard error: 2.698

Table of predicted means for Week

Week 4 8 12

* * *

All values in table missing: due to missing factor combinations in higher order interactions.

Table of predicted means for Strain

Strain 107 2e 316F II PBS

* 53.61 35.77 45.58 *

Standard errors

Strain 107 2e 316F II PBS

* 4.73 4.64 4.64 *

Standard errors

Average: 4.673

Maximum: 4.730

Minimum: 4.644

Table of predicted means for Week.Strain

Strain 107 2e 316F II PBS

Week

4 * 57.70 40.70 40.70 *

8 * 59.25 32.50 47.65 *

12 * 43.89 34.10 48.40 *

Standard errors

Strain 107 2e 316F II PBS

Week

4 * 8.04 8.04 8.04 *

8 * 8.04 8.04 8.04 *

12 * 8.48 8.04 8.04 *

Standard errors

Average: 8.093

Maximum: 8.479

Minimum: 8.044

There is still statistically significant evidence (p=0.028) of differences in the means of the rank densities for different strains, but no evidence of any Week effect, either alone or in interaction with Strain.

1. **Analysis of leucocyte clusters with AFB**

To aid in interpretation of the models of ranked data, we tabulate the means for the unranked data:

Week 4 8

Nobservd Mean Nobservd Mean

Strain

107 10 0.1104 10 0.8241

2e 10 0.0327 10 0.0386

316F 10 0.0067 10 0.0148

II 10 0.0203 10 0.0607

PBS 10 0.0000 10 0.0000

Week 12

Nobservd Mean

Strain

107 9 0.4438

2e 9 0.0000

316F 10 0.0148

II 10 0.0157

PBS 10 0.0000

Because of an unusual distribution of observed values in these data, they have been analysed using the ranks of the data in a Linear Mixed Model. Fitting the model to the MAP groups alone, the output is as follows:

REML variance components analysis

Response variate: LCA/AFB_Rank

Fixed model: Constant + Week + Strain + Week.Strain

Number of units: 118

Residual term has been added to model

Sparse algorithm with AI optimisation

Analysis is subject to the restriction on LCA/AFB_Rank

Residual variance model

Term Factor Model(order) Parameter Estimate s.e.

Residual Identity Sigma2 526.8 72.4

Tests for fixed effects

Sequentially adding terms to fixed model

Fixed term Wald statistic n.d.f. F statistic d.d.f. F pr

Week 17.44 2 8.72 106.0 <0.001

Strain 87.15 3 29.05 106.0 <0.001

Week.Strain 24.41 6 4.07 106.0 0.001

Dropping individual terms from full fixed model

Fixed term Wald statistic n.d.f. F statistic d.d.f. F pr

Week.Strain 24.41 6 4.07 106.0 0.001

*Message: denominator degrees of freedom for approximate F-tests are calculated using algebraic derivatives ignoring fixed/boundary/singular variance parameters.*

Table of effects for Constant

66.30 Standard error: 7.258

Table of effects for Week

Week 4 8 12

0.00 42.70 35.48

Standard errors

Week 4 8 12

0.00 10.26 10.55

Standard errors

Average: 10.40

Maximum: 10.55

Minimum: 10.26

Table of effects for Strain

Strain 107 2e 316F II PBS

0.00 -5.95 -28.35 -15.55 *

Standard errors

Strain 107 2e 316F II PBS

0.00 10.26 10.26 10.26 *

Table of effects for Week.Strain

Strain 107 2e 316F II PBS

Week

4 0.00 0.00 0.00 0.00 *

8 0.00 -46.20 -35.55 -17.20 *

12 0.00 -68.33 -38.58 -37.88 *

Standard errors

Strain 107 2e 316F II PBS

Week

4 0.00 0.00 0.00 0.00 *

8 0.00 14.52 14.52 14.52 *

12 0.00 14.91 14.72 14.72 *

Standard errors

Average: 14.65

Maximum: 14.91

Minimum: 14.52

Table of predicted means for Constant

59.59 Standard error: 2.115

Table of predicted means for Week

Week 4 8 12

* * *

All values in table missing: due to missing factor combinations in higher order interactions.

Table of predicted means for Strain

Strain 107 2e 316F II PBS

92.36 48.23 39.30 58.45 *

Standard errors

Strain 107 2e 316F II PBS

4.27 4.27 4.19 4.19 *

Standard errors

Average: 4.229

Maximum: 4.267

Minimum: 4.190

Table of predicted means for Week.Strain

Strain 107 2e 316F II PBS

Week

4 66.30 60.35 37.95 50.75 *

8 109.00 56.85 45.10 76.25 *

12 101.78 27.50 34.85 48.35 *

Standard errors

Strain 107 2e 316F II PBS

Week

4 7.26 7.26 7.26 7.26 *

8 7.26 7.26 7.26 7.26 *

12 7.65 7.65 7.26 7.26 *

Standard errors

Average: 7.323

Maximum: 7.651

Minimum: 7.258

There is a highly statistically significant (p<0.001) difference in the mean rank densities for different strains, and strong evidence (p=0.001) that the pattern of change over successive weeks is different for different strains. The biggest difference is between the mean for the wildtype strain and all the others. Refitting the model to only the vaccine strains gives:

REML variance components analysis

Response variate: LCA/AFB_Rank

Fixed model: Constant + Week + Strain + Week.Strain

Number of units: 89

Residual term has been added to model

Sparse algorithm with AI optimisation

Analysis is subject to the restriction on LCA/AFB_Rank

Residual variance model

Term Factor Model(order) Parameter Estimate s.e.

Residual Identity Sigma2 433.8 68.6

Tests for fixed effects

Sequentially adding terms to fixed model

Fixed term Wald statistic n.d.f. F statistic d.d.f. F pr

Week 13.58 2 6.79 80.0 0.002

Strain 10.34 2 5.17 80.0 0.008

Week.Strain 7.49 4 1.87 80.0 0.123

Dropping individual terms from full fixed model

Fixed term Wald statistic n.d.f. F statistic d.d.f. F pr

Week.Strain 7.49 4 1.87 80.0 0.123

*Message: denominator degrees of freedom for approximate F-tests are calculated using algebraic derivatives ignoring fixed/boundary/singular variance parameters.*

Table of effects for Constant

55.65 Standard error: 6.586

Table of effects for Week

Week 4 8 12

0.00 -4.00 -30.15

Standard errors

Week 4 8 12

0.00 9.31 9.57

Standard errors

Average: 9.442

Maximum: 9.570

Minimum: 9.314

Table of effects for Strain

Strain 107 2e 316F II PBS

* 0.000 -20.300 -8.600 *

Standard errors

Strain 107 2e 316F II PBS

* 0.000 9.314 9.314 *

Table of effects for Week.Strain

Strain 107 2e 316F II PBS

Week

4 * 0.00 0.00 0.00 *

8 * 0.00 10.65 26.10 *

12 * 0.00 26.65 27.95 *

Standard errors

Strain 107 2e 316F II PBS

Week

4 * 0.00 0.00 0.00 *

8 * 0.00 13.17 13.17 *

12 * 0.00 13.35 13.35 *

Standard errors

Average: 13.26

Maximum: 13.35

Minimum: 13.17

Table of predicted means for Constant

44.78 Standard error: 2.209

Table of predicted means for Week

Week 4 8 12

* * *

All values in table missing: due to missing factor combinations in higher order interactions.

Table of predicted means for Strain

Strain 107 2e 316F II PBS

* 44.27 36.40 53.68 *

Standard errors

Strain 107 2e 316F II PBS

* 3.87 3.80 3.80 *

Standard errors

Average: 3.826

Maximum: 3.872

Minimum: 3.803

Table of predicted means for Week.Strain

Strain 107 2e 316F II PBS

Week

4 * 55.65 35.35 47.05 *

8 * 51.65 42.00 69.15 *

12 * 25.50 31.85 44.85 *

Standard errors

Strain 107 2e 316F II PBS

Week

4 * 6.59 6.59 6.59 *

8 * 6.59 6.59 6.59 *

12 * 6.94 6.59 6.59 *

Standard errors

Average: 6.626

Maximum: 6.943

Minimum: 6.586

There is still statistically significant evidence (p=0.008) of differences in the means of the rank densities for different strains, and evidence of differences in the means for different weeks (p=0.002), but no evidence for the trends over time being different for different strains (p=0.123).

To summarise the means for different strains and weeks, we refit the model without the interaction term:

REML variance components analysis

Response variate: LCA/AFB_Rank

Fixed model: Constant + Week + Strain

Number of units: 89

Residual term has been added to model

Sparse algorithm with AI optimisation

Analysis is subject to the restriction on LCA/AFB_Rank

Residual variance model

Term Factor Model(order) Parameter Estimate s.e.

Residual Identity Sigma2 451.8 69.7

Tests for fixed effects

Sequentially adding terms to fixed model

Fixed term Wald statistic n.d.f. F statistic d.d.f. F pr

Week 13.03 2 6.52 84.0 0.002

Strain 9.93 2 4.96 84.0 0.009

Dropping individual terms from full fixed model

Fixed term Wald statistic n.d.f. F statistic d.d.f. F pr

Week 13.04 2 6.52 84.0 0.002

Strain 9.93 2 4.96 84.0 0.009

*Message: denominator degrees of freedom for approximate F-tests are calculated using algebraic derivatives ignoring fixed/boundary/singular variance parameters.*

Table of effects for Constant

45.69 Standard error: 5.034

Table of effects for Week

Week 4 8 12

0.000 8.250 -11.666

Standard errors

Week 4 8 12

0.000 5.488 5.537

Standard errors

Average: 5.512

Maximum: 5.537

Minimum: 5.488

Table of effects for Strain

Strain 107 2e 316F II PBS

* 0.000 -8.151 9.133 *

Standard errors

Strain 107 2e 316F II PBS

* 0.000 5.537 5.537 *

Table of predicted means for Constant

44.88 Standard error: 2.254

Table of predicted means for Week

Week 4 8 12

46.02 54.27 34.35

Standard errors

Week 4 8 12

3.88 3.88 3.95

Standard errors

Average: 3.903

Maximum: 3.949

Minimum: 3.881

Table of predicted means for Strain

Strain 107 2e 316F II PBS

* 44.55 36.40 53.68 *

Standard errors

Strain 107 2e 316F II PBS

* 3.95 3.88 3.88 *

Standard errors

Average: 3.903

Maximum: 3.949

Minimum: 3.881
